# An equilibrium optimizer slime mould algorithm for inverse kinematics of the 7-DOF robotic manipulator

**DOI:** 10.1038/s41598-022-13516-3

**Published:** 2022-06-08

**Authors:** Shihong Yin, Qifang Luo, Guo Zhou, Yongquan Zhou, Binwen Zhu

**Affiliations:** 1 College of Artificial Intelligence, Guangxi University for Nationalities, Nanning, 530006 China; 2 Department of Science and Technology Teaching, China University of Political Science and Law, Beijing, 102249 China; 3 Guangxi Key Laboratories of Hybrid Computation and IC Design Analysis, Nanning, 530006 China

**Keywords:** Engineering, Mathematics and computing

## Abstract

In order to solve the inverse kinematics (IK) of complex manipulators efficiently, a hybrid equilibrium optimizer slime mould algorithm (EOSMA) is proposed. Firstly, the concentration update operator of the equilibrium optimizer is used to guide the anisotropic search of the slime mould algorithm to improve the search efficiency. Then, the greedy strategy is used to update the individual and global historical optimal to accelerate the algorithm’s convergence. Finally, the random difference mutation operator is added to EOSMA to increase the probability of escaping from the local optimum. On this basis, a multi-objective EOSMA (MOEOSMA) is proposed. Then, EOSMA and MOEOSMA are applied to the IK of the 7 degrees of freedom manipulator in two scenarios and compared with 15 single-objective and 9 multi-objective algorithms. The results show that EOSMA has higher accuracy and shorter computation time than previous studies. In two scenarios, the average convergence accuracy of EOSMA is 10e−17 and 10e−18, and the average solution time is 0.05 s and 0.36 s, respectively.

## Introduction

The inverse kinematics (IK) problem is to determine the joint angle based on the position and posture of the manipulator's end-effector^1^. That is, the purpose is to accurately transfer the end-effector to the desired position and posture^2^. It is one of the most fundamental problems in robot technology and plays an essential role in robot motion control, trajectory planning, and dynamic analysis^3^. However, the IK of redundant manipulators is a complex problem due to nonlinear Equations^4^. The traditional methods for solving inverse kinematics mainly include the analytic method and numerical iteration method^5,6^. The IK problem has an analytical solution for a manipulator that conforms to the Pieper standard. However, with the increase of the types of manipulators, many manipulators do not meet the Pieper standard, such as serial-parallel manipulators driven by cable^7^ and super-redundant serial manipulators^8^. The IK of redundant manipulators may have many group solutions. Still, it is difficult to obtain satisfactory solutions by traditional methods, and the real-time performance is poor. As a result, it is preferable to solve the IK of the complex manipulator using a metaheuristic approach^9^. Metaheuristic algorithm is a random method that is a successful alternative to the precise methods for solving practical optimization problems^10,11^. The advantages of metaheuristics include simplicity of principle, ease of implementation, independence form the problem, and gradient-free characteristics^12^. Many metaheuristic algorithms, which including particle swarm optimization (PSO)^9^, firefly algorithm (FA)^13^, artificial bee colony algorithm (ABC)^14^, and others, have been effectively applied to the IK of robotic manipulators. Although these algorithms have achieved excellent convergence accuracy, they often do not take into account the end-effector's posture, which reduces the complexity of the IK problem and is inconsistent with most practical applications.

Slime mould algorithm (SMA) is an unique metaheuristic algorithm developed by Li et al*.*^15^ in 2020. Due to its capacity to imitate the peculiar oscillatory foraging behavior of slime mould and its remarkable performance, SMA has been effectively applied in a wide variety of fields in less than two years. For example, Abdel-Basset et al*.*^16^ and Ewees et al*.*^17^ applied the improved SMA to feature selection problems; Abdel-Basset et al*.*^18^, Naik et al*.*^19^ and Zhao et al*.*^20^ used hybrid and improved SMA to solve image segmentation problem (ISP); El-Fergany^21^, Kumar et al*.*^22^, Liu et al*.*^23^, Mostafa et al*.*^24^ and Yousri et al*.*^25^ used hybrid and improved SMA to estimate parameters of solar photovoltaic cells, respectively; Agarwal and Bharti^26^ applied improved SMA to the collision-free shortest time path planning of mobile robots; Rizk-Allah et al*.*^27^ proposed a chaos-opposition-enhanced SMA (CO-SMA) to minimize the energy costs of wind turbines at high-altitude sites; Hassan et al*.*^28^ applied improved SMA (ISMA) to efficiently solve economic and emission dispatch (EED) problem with single and dual objectives; Abdollahzadeh et al*.*^29^ proposed a binary SMA to solve the 0–1 knapsack problem; Zubaidi et al*.*^30^ combined SMA and artificial neural network (ANN) for urban water demand prediction; Chen and Liu^31^ combined K-means clustering and chaotic SMA with support vector regression to obtain higher prediction accuracy; Ekinci et al*.*^32^ applied SMA to the power system stabilizer design (PSSD); Wazery et al*.*^33^ combined SMA and K-nearest neighbor for disease classification and diagnosis system; Wei et al*.*^34^ proposed an enhanced SMA in power systems for optimal reactive power dispatch; Premkumar et al*.*^35^ and Houssein et al*.*^36^ developed multi-objective SMA (MOSMA) for solving complicated multi-objective engineering design problems in the real world; Yu et al*.*^37^ proposed an improved SMA (WQSMA) that enhanced the original SMA's robustness by using a quantum rotation gate (QRG) and a water cycle operator. Houssein et al*.*^38^ proposed a hybrid SMA and adaptive guided differential evolution (AGDE) algorithm, which makes a good combination of SMA's exploitation ability and AGDE's exploration ability.

Although SMA has been used in many fields, it has not been applied to the IK problem. SMA, like most metaheuristic algorithms, suffers from diversity loss and premature convergence as a result of an improper balance between exploration and exploitation (weak exploration ability) during the iterative process of addressing difficult optimization problems. In order to improve the searching ability of SMA, the update strategy of equilibrium optimizer (EO) is used to replace the anisotropic operator of SMA to guide the search of slime mould more efficiently. Secondly, the greedy selection strategy is used to preserve the individual historical optimal location and search based on the information of the individual historical optimal to accelerate the algorithm’s convergence. Finally, to increase the possibility of escaping from the local optimal and avoid overcrowding, a random difference mutation operator is added to the algorithm. In EOSMA, the update operator of EO benefits from an appropriate balance of exploration and exploitation, the search operator of SMA is in charge of the main exploitation, and the random difference mutation operator expands the search range of the search agents during iteration while maintaining population diversity. To verify the efficiency of EOSMA in solving the IK problem of complex manipulator, it is compared with slime mould algorithm (SMA)^15^, equilibrium optimizer (EO)^39^, manta ray foraging optimization (MRFO)^40^, marine predators algorithm (MPA)^41^, pathfinder algorithm (PFA)^42^, flower pollination algorithm (FPA)^43^, differential evolution (DE)^44^, gradient-based optimizer (GBO)^45^, teaching–learning-based optimization (TLBO)^46^, Harris hawks optimization (HHO)^47^, improved grey wolf optimizer (IGWO)^48^, hybrid PSO and gravitational search algorithm (PSOGSA)^49^, centroid opposition-based differential evolution (CODE)^50^, multi-trial vector-based differential evolution (MTDE)^51^, self-adaptive spherical search algorithm (SASS)^52^ and the results of previous studies. Then, a multi-objective EOSMA (MOEOSMA) is proposed and compared with MOSMA^35^, multi-objective PSO (MOPSO)^53^, multi-objective MPA (MOMPA)^54^, multi-objective ant lion optimizer (MOALO)^55^, multi-objective dragonfly algorithm (MODA)^56^, multi-objective grey wolf optimizer (MOGWO)^57^, multi-objective multi-verse optimization (MOMVO)^58^, multi-objective salp swarm algorithm (MSSA)^59^, multi-objective evolutionary algorithm based on decomposition (MOEA/D)^60^ on the IK problem of a 7 degrees of freedom (DOF) manipulator. This paper's primary contributions are as follows:

1. A hybrid EOSMA was developed to enhance the algorithm's search capability and balance exploration and exploitation;
2. By introducing the archiving mechanism of non-dominated solutions, a multi-objective variant of EOSMA (MOEOSMA) was developed;
3. EOSMA and MOEOSMA were applied to the IK of the redundant manipulator to validate the algorithm's performance and broaden its application range;
4. The influence of end-effector posture on the IK problem was investigated in order to provide a reference for relevant researchers.

The remainder of this work is structured as follows. Section “Related works” provides a synopsis of relevant works in the literature. Section “Preliminaries” introduces the SMA and EO algorithms, as well as the basic notions of multi-objective optimization. Section “The proposed EOSMA algorithm” describes the implementation steps of the EOSMA and MOEOSMA in detail. Section “Kinematics analysis of manipulator” presents the manipulator’s kinematics equation. The fitness function for the IK problem is defined in Sect. “EOSMA for inverse kinematics”. Section “Experimental results and discussions” reports and discusses the experimental results. Finally, Sect. “Conclusions and future directions” concludes the paper.

## Related works

Inverse kinematics is a fundamental problem of robot technology, which plays a crucial role in robot trajectory planning, motion control, and dynamics analysis^61^. Due to the inverse kinematics equation being highly nonlinear, the traditional algorithm takes a long time to solve, and it is difficult to obtain ideal results. Therefore, previous researchers developed a variety of metaheuristic algorithms to address the IK problem of robotic manipulators. Huang et al*.*^62^ employed PSO to tackle the IK problem of a 7-DOF robotic manipulator; Ram et al*.*^63^ used a bidirectional PSO approach to address the IK problem caused by manipulator position shift; Adly et al*.*^64^ proposed single-objective and multi-objective versions of improved PSO, and verified the performance of the algorithm on 5-DOF and 7-DOF robotic manipulators; Ayyıldız and Çetinkaya^65^ solved IK of a 4-DOF serial robotic manipulator using GA, PSO, QPSO, and GSA. According to the results, QPSO has the best problem-solving performance; Dereli and Köker^9^ applied QPSO to solve the IK of 7-DOF serial manipulator and compared it with FA, PSO, and ABC. The results show that QPSO has higher solving accuracy and shorter calculation time than the contrast algorithm; Liu et al*.*^66^ proposed a parallel learning PSO (PLPSO) to solve the IK problem and verified the practicability and feasibility of the algorithm on UR5 manipulator; Dereli and Köker^67^ proposed a RDV-PSO that combines golf ball movements and PSO, and applied it to the IK solution of 7-DOF manipulator; Momani et al*.*^68^ applied the traditional GA and the continuous GA to the IK problem respectively, and the results showed that the continuous GA was superior to the traditional GA in all aspects; López-Franco et al*.*^69^ applied DE to the IK of the manipulator. Simulation and experimental results show the applicability of this method; Rokbani et al*.*^70^ applied FA to the IK problem and tested it on a three-link articulated planar system, and conducted a statistical analysis on the convergence and solution quality of 100 tests; Dereli and Köker^13^ applied FA to the IK problem of a 7-DOF redundant manipulator and compared it with PSO and ABC; Çavdar and Milani^71^ proposed a method for solving IK of a robot manipulator based on improved ABC, and the results illustrate that the proposed algorithm outperforms PSO and HS in positioning accuracy and solving time; El-Sherbiny et al*.*^72^ proposed K-ABC, which used different parameters in the process of updating food sources, and then used K-ABC to calculate the IK of a 5-DOF manipulator. Dereli and Köker^73^ proposed an ABC for solving the IK of the 7-DOF manipulator; Zhang and Xiao^14^ proposed a CPABC algorithm based on ABC to solve the IK of 7-DOF manipulator. The CPABC utilized chaotic mapping to optimize the population distribution of the initial food source and avoided local optimum; Dereli^74^ used the modified GWO, FPD-GWO, to solve the IK problem and compared it with GWO. The results reveal that FPD-GWO has a significantly higher convergence accuracy than GWO; Dereli^75^ proposed an modified WOA, ASI-WOA, which avoided the problems of sluggish convergence speed and frequent falling into local optimum, and evaluated the performance of ASI-WOA on the IK problem; Toz^76^ proposed a vortex search algorithm based on chaotic mapping (CVS), and verified the performance of CVS on a 6-DOF series manipulator; Wu et al*.*^77^ proposed an algorithm that combines the parameterization method with the T-IK method to address the IK problem in the position domain of redundant manipulators, and they tested the T-IK algorithm on an 8-DOF tunnel shotcrete robot. However, the posture of the end-effector is usually not considered in previous studies when solving IK problems, and the performance of solving accuracy, stability, and real-time performance of algorithms need to be further improved.

## Preliminaries

### Slime mould algorithm

Slime mould algorithm (SMA) is a metaheuristic algorithm developed by Li et al*.*^15^ that is inspired by slime mould's peculiar oscillatory foraging behavior. Slime mould can explore for food sources based on the odor concentration of food in the air during foraging. In this process, SMA mainly simulates three different morphologies of slime mould foraging: (1) When $rand < z$, the contraction pattern of slime mould is unstable and becomes anisotropic, which can be searched anywhere in the search space; (2) When $r < p$, slime mould begins to form thick vein-like tube along the radius; (3) When $r \ge p$, the contractile morphology of slime mould no longer changes over time, and the vascular structure disappears, as shown in Eq. (1).

1 $$ \overrightarrow {{X^{ * } }} = \left\{ {\begin{array}{*{20}l} {rand \cdot (UB - LB) + LB} \hfill & {rand < z} \hfill \\ {\overrightarrow {{X_{b} }} + \overrightarrow {vb} \cdot \left( {\overrightarrow {W} \cdot \overrightarrow {{X_{A} }} - \overrightarrow {{X_{B} }} } \right)} \hfill & {r < p} \hfill \\ {\overrightarrow {vc} \cdot \overrightarrow {X} } \hfill & {r \ge p} \hfill \\ \end{array} } \right. $$

where $\overrightarrow {W}$ is the search agent's fitness weight, $\overrightarrow {vb}$ is a random number vector in $[ - a,a]$, and $\overrightarrow {vc}$ declines linearly from 1 to 0, and $\overrightarrow {{X_{b} }}$ is the best location of the current iteration. $\overrightarrow {{X_{A} }}$ and $\overrightarrow {{X_{B} }}$ are two locations selected at random from the population. The value of $p$ is calculated as Eq. (2).

2 $$ p = \tanh |S(i) - DF| $$

where $S$ signifies the fitness of the search agents and $DF$ denotes the best fitness of all iterations. The value of $a$ in the range of $\overrightarrow {vb}$ is calculated as Eq. (3).

3 $$ a = {\text{atanh}} \left( {1 - {t \mathord{\left/ {\vphantom {t {\max \_t}}} \right. \kern-\nulldelimiterspace} {\max \_t}}} \right) $$

The $\overrightarrow {W}$ is calculated as Eq. (4).

4 $$ \overrightarrow {W(SIdx(i))} = \left\{ {\begin{array}{*{20}l} {1 + r \cdot \log \left( {\frac{bF - S(i)}{{bF - wF}} + 1} \right)} \hfill & {i < \frac{N}{2}} \hfill \\ {1 - r \cdot \log \left( {\frac{bF - S(i)}{{bF - wF}} + 1} \right)} \hfill & {others} \hfill \\ \end{array} } \right. $$

5 $$ SIdx = sort(S) $$

where $N$ represents the population size, $r$ is a random number vector in the range [0, 1], $bF$ is the best fitness in the current iteration, and $wF$ is the poorest fitness, $SIdx$ represents the result of the ascending order of fitness.

### Characteristics of SMA

SMA has the advantages of simple principle, low time complexity, and fast convergence speed. The elite strategy, ranking mechanism, and archiving mechanism are not adopted. All search individuals simply and equally choose to be close to or away from the best food source $\overrightarrow {{X_{b} }}$. The location $\overrightarrow {X}$ is updated based on the currently obtained optimal location $\overrightarrow {{X_{b} }}$, and the population of slime mould is continuously guided to converge to the optimal location rapidly. As a result, SMA's exploitation ability outperforms that of exploration, and it is easy to fall into a local optimum. In addition, from Eq. (1), it can be seen that the performance of SMA mainly comes from the oscillatory foraging process of simulating slime mould to form vein-like tubes. In fact, for most real-world application problems, the first operator and the third operator of Eq. (1) are inefficient. The first operator only searches randomly, and the third operator will guide the slime mould to converge to the origin, which reduces the search efficiency. Therefore, the update operator of SMA will be simplified and improved in this paper. Please refer to^15^ for the detailed steps and pseudo code of the SMA.

### Equilibrium optimizer

The equilibrium optimizer (EO) is a physically-based metaheuristic algorithm developed by Faramarzi et al*.* in 2020 that is inspired by mass balance of controlled volume and can estimate dynamic and equilibrium states simultaneously^39^. The mass balance equation describes the physical process of mass entering, exiting, and generating in the control volume^78^. In EO, search agents update their concentration (location) at random in order to find some genius particles known as equilibrium candidates in order to attain the final equilibrium state as the global optimal. Equation (6) shows the updating formula.

6 $$ \overrightarrow {C} = \overrightarrow {{C_{eq} }} + \overrightarrow {F} \left( {\overrightarrow {C} - \overrightarrow {{C_{eq} }} } \right) + {{\left( {1 - \overrightarrow {F} } \right)\overrightarrow {G} } \mathord{\left/ {\vphantom {{\left( {1 - \overrightarrow {F} } \right)\overrightarrow {G} } {\left( {\overrightarrow {\lambda } \cdot V} \right)}}} \right. \kern-\nulldelimiterspace} {\left( {\overrightarrow {\lambda } \cdot V} \right)}} $$

where $\overrightarrow {C}$ is the current solution, $\overrightarrow {{C_{eq} }}$ is a randomly selected solution from the equilibrium pool, $\overrightarrow {F}$ is an adaptive parameter, $\overrightarrow {G}$ is the mass generation rate, $\overrightarrow {\lambda }$ is a random number vector in [0, 1], and $V = 1$ signifies the unit volume. There are five candidate solutions in the equilibrium pool. Four are the best candidate solutions found so far, and another is the average concentration (center location) of these four candidate solutions, as shown in Eq. (7).

7 $$ \overrightarrow {{C_{eq,pool} }} = \left\{ {\overrightarrow {{C_{eq,1} }} ,\overrightarrow {{C_{eq,2} }} ,\overrightarrow {{C_{eq,3} }} ,\overrightarrow {{C_{eq,4} }} ,\overrightarrow {{C_{eq,ave} }} } \right\} $$

The $\overrightarrow {F}$ is adaptively adjusted according to Eq. (8).

8 $$  \begin{aligned}   \overrightarrow {F}  =  & a_{1}  \cdot sign\left( {\overrightarrow {r}  - 0.5} \right) \cdot \left( {e^{{ - \overrightarrow {\lambda } t_{1} }}  - 1} \right) \\    t_{1}  =  & \left( {1 - {t \mathord{\left/ {\vphantom {t {\max \_t}}} \right. \kern-\nulldelimiterspace} {\max \_t}}} \right)^{{\left( {a_{2}  \cdot \tfrac{t}{{\max \_t}}} \right)}}  \\  \end{aligned}    $$

where $a_{1} \in [1,2]$ and $a_{2} \in [1,2]$ control the exploration and exploitation, respectively. The larger $a_{1}$ is, the stronger the exploration ability is, and the larger $a_{2}$ is, the stronger the development ability is, and vice versa. $sign$ represents the symbolic function. $\overrightarrow {r}$ and $\overrightarrow {\lambda }$ are vectors of random numbers in [0, 1]. The $\overrightarrow {G}$ is calculated by Eq. (9).

9 $$ \overrightarrow {G} = \left\{ {\begin{array}{*{20}l} {0.5r_{1} \left( {\overrightarrow {{C_{eq} }} - \overrightarrow {\lambda } \overrightarrow {C} } \right)\overrightarrow {F} } \hfill & {r_{2} \ge GP} \hfill \\ 0 \hfill & {r_{2} < GP} \hfill \\ \end{array} } \right. $$

where $r_{1}$ and $r_{2}$ are random numbers in [0, 1] and $GP = 0.5$ is the generation probability. More detailed steps and pseudo-code for EO are given in^39^.

### The basic notions of multi-objective optimization

Multi-objective optimization needs to optimize two or more objective functions simultaneously and cannot balance them explicitly; that is, there is no optimal solution that meets all objectives at once. Without loss of generality, multi-objective optimization can be expressed as the following optimization problem^79^:

10 $$ \begin{gathered} {\text{Minimize: }}F(\vec{x}) = \left( {f_{1} (\vec{x}),f_{2} (\vec{x}), \ldots ,f_{M} (\vec{x})} \right)^{{\text{T}}} \hfill \\ {\text{s.t}}{. : }g_{i} (\vec{x}) \le 0,i = 1,2, \ldots ,m \hfill \\ \, h_{i} (\vec{x}) = 0,i = 1,2, \ldots ,n \hfill \\ \, \vec{x} = (x_{1} ,x_{2} , \ldots ,x_{Dim} ) \hfill \\ \, L_{i} \le x_{i} \le U_{i} ,i = 1,2, \ldots ,Dim \hfill \\ \end{gathered} $$

where $M$ represents the number of sub-objectives, $m$ denotes the number of inequality constraints, and $n$ denotes the number of equality constraints, $Dim$ represents the dimension of decision variables, $[L,U]$ represents the search range of decision variables.

There is usually no optimal solution for multi-objective optimization problems that minimizes all sub-objectives simultaneously. In this scenario, utilizing arithmetic relation operators to compare different solutions is not possible. In the multi-objective search space, we can compare the two search agents using Pareto optimal dominance^56^. The following are the definitions of Pareto dominance and Pareto optimality:

#### Definition 1^80^

(*Pareto dominance*). Assume there are two vectors, $\vec{x}$ and $\vec{y}$. If and only if the following criteria are met, vector $\vec{x}$ dominates $\vec{y}$ (expressed as $\vec{x} \succ \vec{y}$):

11 $$ \forall i \in \{ 1,2, \ldots ,M\} :f_{i} (\vec{x}) \le f_{i} (\vec{y}) \wedge \exists i \in \{ 1,2, \ldots ,M\} :f_{i} (\vec{x}) < f_{i} (\vec{y}) $$

according to Eq. (11), a solution vector $\vec{x}$ is superior to another $\vec{y}$ if it has better or equal values on all objectives and better values on at least one of them.

#### Definition 2^80^

(*Pareto optimality*). If and only if the following criteria are met, a solution vector $\vec{x} \in D$ is said to be Pareto optimal:

12 $$ \neg \exists \vec{y} \in D:\vec{y} \succ \vec{x} $$

where $D$ denotes the decision space. According to Eq. (12), if no other solution vector in decision space $D$ is superior to $\vec{x}$, then $\vec{x}$ is considered the Pareto optimal solution.

#### Definition 3^80^

(*Pareto optimal set*). The Pareto optimal set (PS) is a set that contains all non-dominated solutions to a given problem:

13 $$ {\text{PS}} = \{ \vec{x}|\neg \exists \vec{y} \in D:\vec{y} \succ \vec{x}\} $$

#### Definition 4^80^

(*Pareto optimal front*). The Pareto optimal front (PF) is the mapping set of the PS on the objective space, and its expression is as follows:

14 $$ {\text{PF}} = \{ F(\vec{x})|\vec{x} \in PS\} $$

## The proposed EOSMA algorithm

### The EOSMA for single-objective problems

In EOSMA, the following improvement strategies are mainly adopted: (1) The individual and global historical optimal of PSO are introduced^81^. The individual historical optimal is preserved by greedy selection and memory mechanism. In the update operator of SMA, the individual and global historical optimal are used to update, to accelerate the algorithm’s convergence; (2) The concentration update operator of EO is used to replace the less efficient anisotropic search operator in SMA to balance the concentration of slime mould in all directions and improve the search efficiency of the algorithm; (3) The random difference mutation operator is introduced. After the location update, the mutation mechanism is employed to improve the algorithm's exploration ability, helping it to escape from the local optimum and avoid premature convergence; (4) The boundary checking of the algorithm is improved, and the solution vector beyond the search boundary is updated to the midpoint of the current solution to the search boundary to avoid the invalid search. Therefore, the location update formula of EOSMA is shown in Eq. (15).

15 $$ \overrightarrow {X(t + 1)} = \left\{ {\begin{array}{*{20}l} {\overrightarrow {{X_{eq} (t)}} + \left( {\overrightarrow {pBest(t)} - \overrightarrow {{X_{eq} (t)}} } \right) \cdot \overrightarrow {F} + {{\overrightarrow {G} \cdot \left( {1 - \overrightarrow {F} } \right)} \mathord{\left/ {\vphantom {{\overrightarrow {G} \cdot \left( {1 - \overrightarrow {F} } \right)} {\left( {\overrightarrow {\lambda } \cdot V} \right)}}} \right. \kern-\nulldelimiterspace} {\left( {\overrightarrow {\lambda } \cdot V} \right)}}} \hfill & {rand < z} \hfill \\ {\overrightarrow {gBest(t)} + \overrightarrow {vb} \cdot \left( {\overrightarrow {W} \cdot \overrightarrow {{pBest_{A} (t)}} - \overrightarrow {{pBest_{B} (t)}} } \right)} \hfill & {others} \hfill \\ \end{array} } \right. $$

where $\overrightarrow {{X_{eq} }}$ is a randomly selected solution from the equilibrium pool, $\overrightarrow {X}$ is the location of the search agents, $\overrightarrow {gBest}$ is the best location found so far, $\overrightarrow {{pBest_{A} }}$ and $\overrightarrow {{pBest_{B} }}$ are two location vectors randomly selected from the individual historical optimal, $z = 0.5$ is the parameter of the hybrid algorithm obtained by experiments, and the meaning of the remaining parameters are the same as in EO and SMA.

In order to improve the exploration ability of the algorithm and the probability of escaping from the local optimum, the search agents execute the random difference mutation strategy after updating by Eq. (15). The mathematical model of the mutation operator is shown in Eq. (16).

16 $$ \overrightarrow {X(t + 1)} = \overrightarrow {{pBest_{R1} (t)}} + SF \cdot \left( {\overrightarrow {{pBest_{R2} (t)}} - \overrightarrow {{pBest_{R3} (t)}} } \right) $$

where $SF$ is a random number taking value in [0.3, 0.6], $R1,R2,R3$ are three random integer vectors, the element takes value in [1, *N*], and *N* represents the population size.

After the search agent location is updated, check the solution to ensure it is within the search range. For the solution vector beyond the search range, the usual practice is to pull it back to the boundary. In this way, it is easy to produce invalid searches and reduce search efficiency. In EOSMA, the boundaries are checked by Eq. (17).

17 $$ X_{i,j} (t + 1) = \left\{ {\begin{array}{*{20}l} {{{\left( {X_{i,j} (t) + UB} \right)} \mathord{\left/ {\vphantom {{\left( {X_{i,j} (t) + UB} \right)} 2}} \right. \kern-\nulldelimiterspace} 2}} \hfill & {X_{i,j} (t + 1) > UB} \hfill \\ {{{\left( {X_{i,j} (t) + LB} \right)} \mathord{\left/ {\vphantom {{\left( {X_{i,j} (t) + LB} \right)} 2}} \right. \kern-\nulldelimiterspace} 2}} \hfill & {X_{i,j} (t + 1) < LB} \hfill \\ {X_{i,j} (t + 1)} \hfill & {others} \hfill \\ \end{array} } \right. $$

Finally, after each fitness evaluation, the individual historical optimal location is updated using the greedy strategy, as shown in Eq. (18).

18 $$ \overrightarrow {{pBest_{i} (t + 1)}} = \left\{ {\begin{array}{*{20}l} {\overrightarrow {{X_{i} (t + 1)}} } \hfill & {S\left( {\overrightarrow {{X_{i} (t + 1)}} } \right) < S\left( {\overrightarrow {{pBest_{i} (t)}} } \right)} \hfill \\ {\overrightarrow {{pBest_{i} (t)}} } \hfill & {others} \hfill \\ \end{array} } \right. $$

**Figure 1:**
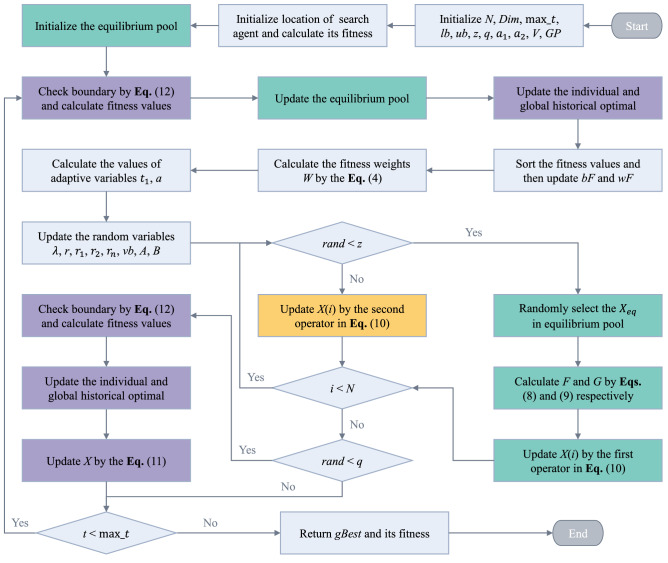
Flow chart of the EOSMA.


In EOSMA, using the $\overrightarrow {{X_{eq} }}$ randomly selected in the equilibrium pool to update the location is equivalent to introducing a GWO-like hierarchical mechanism^12^. Therefore, compared with SMA, EOSMA introduces a greedy selection strategy, hierarchical partitioning mechanism, differential mutation mechanism, and boundary checking strategy. Greedy selection and boundary checking strategy enhance the exploitation ability, and hierarchical partitioning and differential mutation mechanism enhance the exploration ability. As a result, the exploration and exploitation abilities of EOSMA are improved compared with EO and SMA. Figure 1 shows the flowchart of EOSMA, and Algorithm 1 presents its pseudo-code.

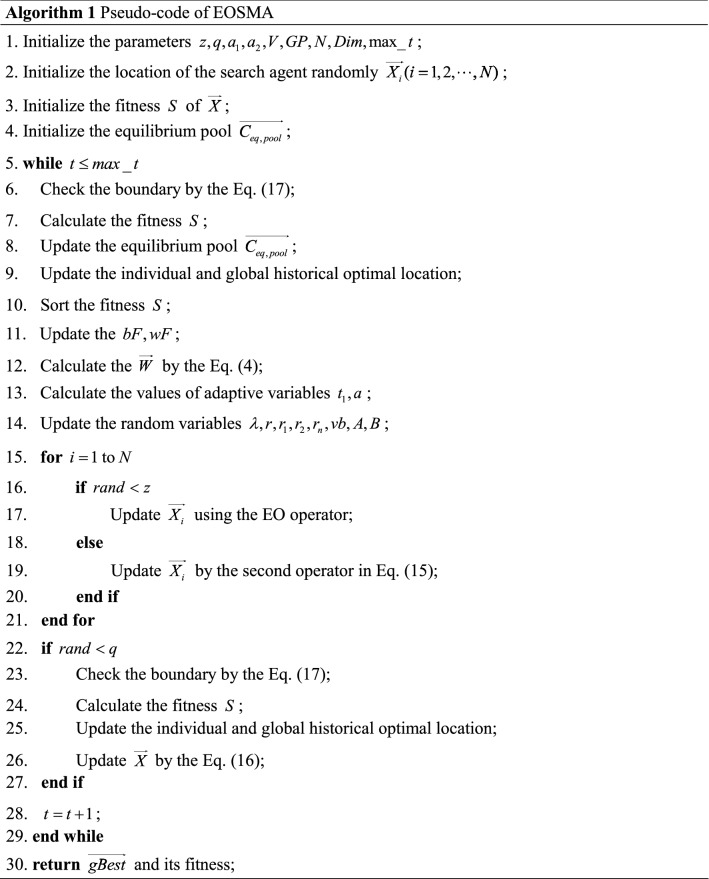


### The EOSMA for multi-objective problems (MOEOSMA)

Two components were added to EOSMA to transform it to a multi-objective version. The first component is an archive that retains all of the Pareto optimal solutions discovered that so far. The second component is a technique for ranking Pareto optimal solutions based on congestion metrics, which updates the equilibrium pool.

The archive is used to store and retrieve PS and PF found so far, and its capacity is the same as the population size. The location update operator of the search agent is the same as EOSMA, but the food source (optimal location) is selected from the archive. An archive updating approach similar to that employed in MOPSO^82^ is used to obtain a well-distributed PF. The archive always collects Pareto optimal solutions from the current population and updates them through the following steps:

1. Combine the new solutions from each iteration with the previous Pareto optimal solutions from the archive, and then check the combined solutions If a solution is not dominated by other solutions, added it to the archive; Otherwise, discard it;
2. Check whether the same solution still exists in the archive, and then remove it;
3. The solutions in the archives are graded based on congestion. The less congested the area, the more important the solutions, and vice versa.
4. If the number of solutions in the archive exceeds the capacity of the archive, the roulette selection method is used to remove the solution with higher congestion;
5. Re-rank the solutions in the archive based on congestion.

**Figure 2:**
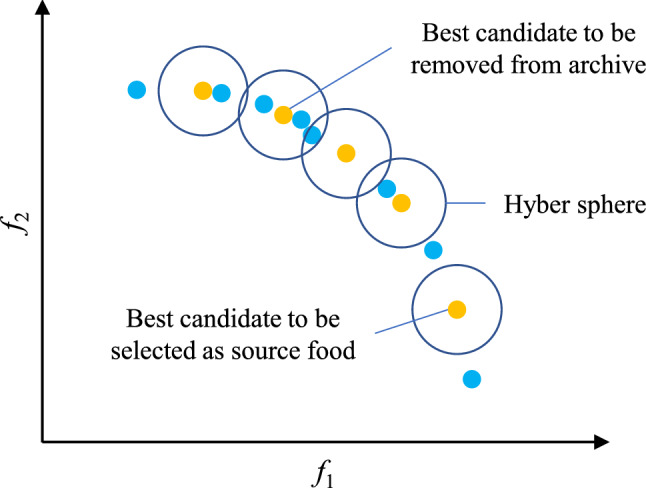
Model of selecting a food source or eliminating a solution from the archive.

All solutions stored in the archive obtained according to the above update rules will dominate other solutions in the population. The conceptual model of congestion level is shown in Fig. 2. A hypersphere with a radius of $\overrightarrow {dr}$ is defined, and the number of solutions in the hypersphere is taken as the congestion level of the solutions, centering on the fitness of each solution. The calculation formula of distance radius $\overrightarrow {dr}$ is Eq. (19).

19 $$ \overrightarrow {dr} = \frac{{\overrightarrow {max} - \overrightarrow {min} }}{Archivesize} $$

where $\overrightarrow {max}$ and $\overrightarrow {min}$ are two vectors that store the maximum and minimum fitness of each objective, respectively, and $Archivesize$ is the archive size^54^.

The multi-objective optimization approach relies on convergence and coverage to obtain the Pareto optimal solution. The convergence is mainly determined by the performance of EOSMA, and the coverage is mainly determined by the archive update rules. As can be seen from Fig. 2, there are more non-dominant solutions near the solutions with higher congestion levels. In order to improve coverage of PF, the solutions with higher congestion levels should be removed preferentially, while the solutions with lower congestion levels need to be preserved vigorously. If the number of non-dominant solutions exceeds the archive capacity, the probability that each solution is removed is calculated using Eq. (20).

20 $$ P_{i} = \frac{{N_{i} }}{C} $$

where $P_{i}$ defines the probability of selecting the *i*-th non-dominated solution, $C$ means the cumulative sum of the congestion levels of all non-dominated solutions, and $N_{i}$ denotes the congestion level of the *i*-th non-dominated solution.

**Figure 3:**
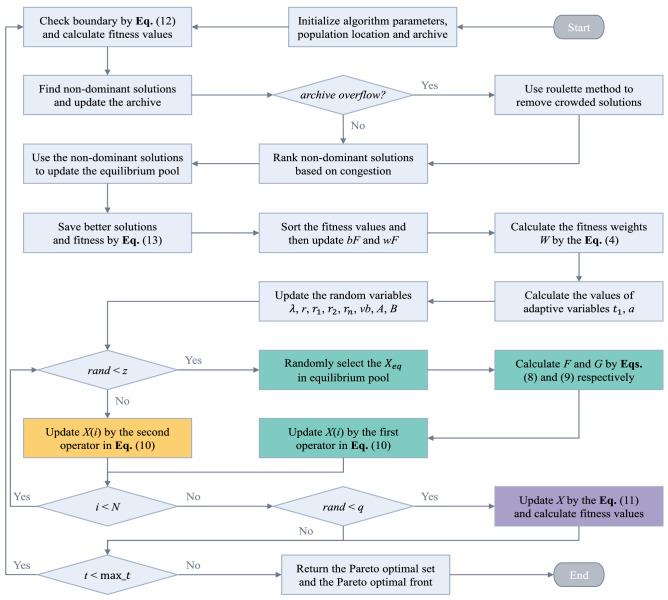
Flow chart of the MOEOSMA.


The equilibrium pool maintains multiple optimal solutions discovered thus far, which broadens the algorithm's search range and improves EOSMA's global search capability. The fitness of search agents can be directly compared for single-objective optimization, and the search agent with the best fitness can be selected and put into the equilibrium pool. For multi-objective optimization, MOEOSMA's archive stores the non-dominant solutions of the current iteration. The solutions with the lowest congestion level can be regarded as the best food source. Therefore, the solution with the lowest congestion level in the archive is put into the equilibrium pool. Each iteration randomly selects a solution in the equilibrium pool as the global optimal location $\overrightarrow {gBest}$ in Eq. (15). It is worth noting that there are 5 solutions in the equilibrium pool of EOSMA, while the number of solutions in the equilibrium pool of MOEOSMA varies. In addition, unlike many heuristic algorithms, SMA needs to sort the fitness during each iteration to evaluate individual fitness weight. Due to the individual fitness of several objectives cannot be compared simultaneously in multi-objective optimization, this work used a rotation sorting approach to estimate the individual fitness weight of slime mould, as shown in Eq. (21).

21 $$ O_{i} = rem(t,M) + 1 $$

where $O_{i}$ signifies the fitness of the *i*-th objective function selected for sorting, $t$ signifies the number of current iterations, and $M$ signifies the number of problem objectives. Figure 3 shows MOEOSMA's flow chart, and Algorithm 2 presents the pseudo-code.

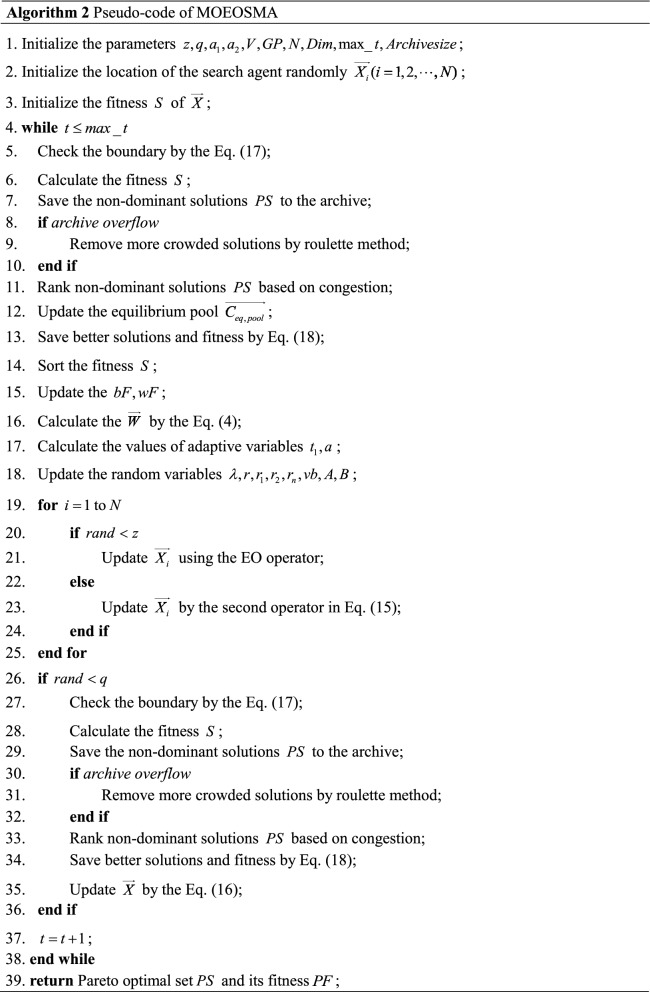


### Complexity analysis

EOSMA comprises sub-components: population initialization, fitness evaluation, greedy selection, fitness sorting, fitness weight update, equilibrium pool update, search agent location update, and mutation operator. The computational complexity of initialization is $O(N * Dim)$, the time complexity of greedy selection and equilibrium pool update are $O(N)$, the computational complexity of fitness weight update, location update, and mutation operation are all $O(N * Dim)$, and the computational complexity of fitness sorting is $O(N * \log N)$. Assuming that the time complexity of the fitness evaluation function is $O(F)$, the time complexity of EOSMA is $O\left( {\max \_t * \left( {N * Dim + N * \log N + F} \right)} \right)$, where $N$ denotes population size, $Dim$ denotes problem dimensionality, $F$ denotes the time to compute the fitness function once, and $\max \_t$ denotes the maximum number of iterations of the algorithm. EOSMA's space complexity is $O(N * Dim)$.

MOEOSMA extends EOSMA components with the archive update operator. It has a time complexity of $O(N_{A}^{2} * M)$, where $N_{A}$ is the archive capacity and $M$ is the number of targets. As a result, MOEOSMA's time complexity is $O\left( {\max \_t * \left( {N_{A}^{2} * M + N * Dim + N * \log N + F} \right)} \right)$. MOEOSMA has the same space complexity as EOSMA, which is $O(N * Dim)$.

## Kinematics analysis of manipulator

**Figure 4:**
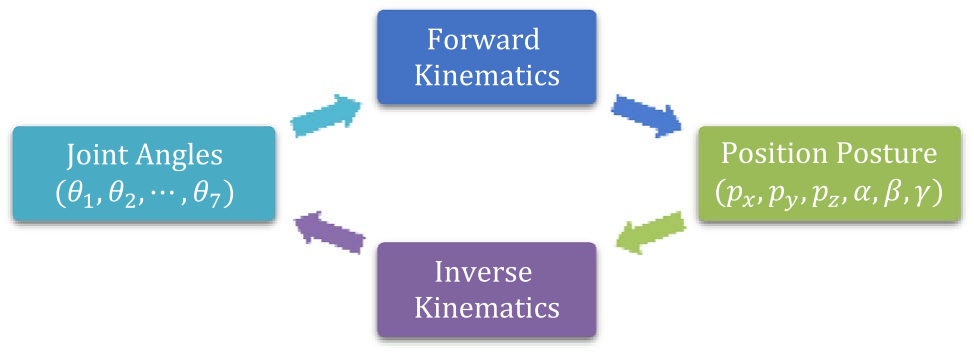
Kinematics analysis of the robotic manipulator.

As illustrated in Fig. 4, the robotic manipulator's kinematics analysis includes forward kinematics (FK) analysis and inverse kinematics (IK) analysis. FK calculates the end-effector's position and posture based on the joint angle vector, and IK calculates the matching joint angle vector based on the position and posture.

**Figure 5:**
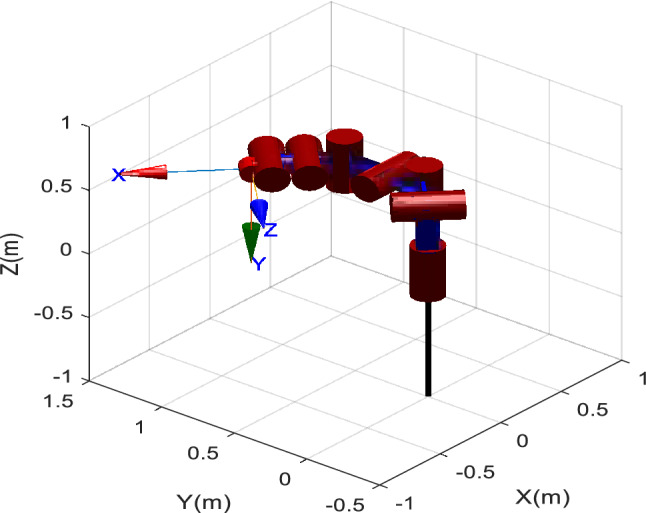
The structure of the 7-DOF robotic manipulator.

IK is a fundamental problem in robotics, which plays an important role in motion control, and trajectory planning^61^. For the manipulator that meets the Pieper standard, the analytical method can be used to solve it. Still, for the more general manipulator, the analytical method cannot be used to solve it, especially for the manipulator with the offset wrist^66^. The manipulator with 7-DOF has been widely used in industry because of its easy obstacle avoidance, flexible movement, and working in a large space^9^. This work uses the previously studied 7-DOF series robotic manipulator^9,74,75^ as a test instance to validate the effectiveness and efficiency of the proposed EOSMA. The structure of the manipulator is shown in Fig. 5, which is composed of 7 rotating joints and 6 connecting rods in series, and the end-effector has an offset of 5 cm. Therefore, the structure of the manipulator does not meet the Pieper standard, and it is difficult to obtain its IK equation by the analytical method.

The forward kinematics model needs to be established before studying the inverse kinematics of the manipulator. Denavit-Hartenberg (DH) parameters can uniquely determine the structure of manipulator and are widely used in FK modeling of robotic manipulator^66^. Table 1 lists the DH parameters of the manipulator studied in this paper, where $a_{i} ,\alpha_{i} ,d_{i} ,\theta_{i}$ represent the length of the connecting rod, the torsion angle of the connecting rod, the offset of the connecting rod, and the joint angle, respectively.

**Table 1:** DH parameters of the 7-DOF robotic manipulator^9^.

Joint	$a_{i} ({\text{m}})$	$\alpha_{i} ({\text{rad}})$	$d_{i} ({\text{m}})$	$\theta_{i} ({\text{rad}}) \,$
1	0	$- {\pi \mathord{\left/ {\vphantom {\pi 2}} \right. \kern-\nulldelimiterspace} 2}$	$l_{1} = 0.5$	$- \pi < \theta_{1} < \pi$
2	$l_{2} = 0.2$	${\pi \mathord{\left/ {\vphantom {\pi 2}} \right. \kern-\nulldelimiterspace} 2}$	0	$- {\pi \mathord{\left/ {\vphantom {\pi 2}} \right. \kern-\nulldelimiterspace} 2} < \theta_{2} < {\pi \mathord{\left/ {\vphantom {\pi 6}} \right. \kern-\nulldelimiterspace} 6}$
3	$l_{3} = 0.25$	$- {\pi \mathord{\left/ {\vphantom {\pi 2}} \right. \kern-\nulldelimiterspace} 2}$	0	$- {\pi \mathord{\left/ {\vphantom {\pi 2}} \right. \kern-\nulldelimiterspace} 2} < \theta_{3} < {{2\pi } \mathord{\left/ {\vphantom {{2\pi } 3}} \right. \kern-\nulldelimiterspace} 3}$
4	$l_{4} = 0.3$	${\pi \mathord{\left/ {\vphantom {\pi 2}} \right. \kern-\nulldelimiterspace} 2}$	0	$- {\pi \mathord{\left/ {\vphantom {\pi 2}} \right. \kern-\nulldelimiterspace} 2} < \theta_{4} < {\pi \mathord{\left/ {\vphantom {\pi 2}} \right. \kern-\nulldelimiterspace} 2}$
5	$l_{5} = 0.2$	$- {\pi \mathord{\left/ {\vphantom {\pi 2}} \right. \kern-\nulldelimiterspace} 2}$	0	$- {\pi \mathord{\left/ {\vphantom {\pi 2}} \right. \kern-\nulldelimiterspace} 2} < \theta_{5} < {\pi \mathord{\left/ {\vphantom {\pi 2}} \right. \kern-\nulldelimiterspace} 2}$
6	$l_{6} = 0.2$	0	0	$- {\pi \mathord{\left/ {\vphantom {\pi 2}} \right. \kern-\nulldelimiterspace} 2} < \theta_{6} < {\pi \mathord{\left/ {\vphantom {\pi 2}} \right. \kern-\nulldelimiterspace} 2}$
7	$l_{7} = 0.1$	0	$d_{7} = 0.05$	$- {\pi \mathord{\left/ {\vphantom {\pi 6}} \right. \kern-\nulldelimiterspace} 6} < \theta_{7} < {\pi \mathord{\left/ {\vphantom {\pi 2}} \right. \kern-\nulldelimiterspace} 2}$

The FK model of the manipulator is established using the standard DH parameter method, and the homogeneous transformation matrix of the single joint is presented in Eq. (22)^5^.

22 $$ {}_{i - 1}^{i} T = \left[ {\begin{array}{*{20}c} {c\theta_{i} } & { - s\theta_{i} \cdot c\alpha_{i} } & {s\theta_{i} \cdot s\alpha_{i} } & {a_{i} \cdot c\theta_{i} } \\ {s\theta_{i} } & {c\theta_{i} \cdot c\alpha_{i} } & { - c\theta_{i} \cdot s\alpha_{i} } & {a_{i} \cdot s\theta_{i} } \\ 0 & {s\alpha_{i} } & {c\alpha_{i} } & {d_{i} } \\ 0 & 0 & 0 & 1 \\ \end{array} } \right] $$

where ${}_{i - 1}^{i} T$ is the homogeneous transformation matrix of joint $i - 1$ to $i$, $s\theta_{i}$ and $c\theta_{i}$ stand in for $\sin (\theta_{i} )$ and $\cos (\theta_{i} )$, respectively.

By substituting each row of data in Table 1 into Eq. (22), the homogeneous transformation matrix of each joint can be obtained, as shown in Eq. (23).

23 $$ \begin{gathered} {}_{0}^{1} T{ = }\left[ {\begin{array}{*{20}c} {c\theta_{1} } & 0 & { - s\theta_{1} } & 0 \\ {s\theta_{1} } & 0 & {c\theta_{1} } & 0 \\ 0 & { - 1} & 0 & {l_{1} } \\ 0 & 0 & 0 & 1 \\ \end{array} } \right]; \, {}_{1}^{2} T{ = }\left[ {\begin{array}{*{20}c} {c\theta_{2} } & 0 & {s\theta_{2} } & {l_{2} c\theta_{2} } \\ {s\theta_{2} } & 0 & { - c\theta_{2} } & {l_{2} s\theta_{2} } \\ 0 & 1 & 0 & 0 \\ 0 & 0 & 0 & 1 \\ \end{array} } \right]; \, \hfill \\ {}_{2}^{3} T{ = }\left[ {\begin{array}{*{20}c} {c\theta_{3} } & 0 & { - s\theta_{3} } & {l_{3} c\theta_{3} } \\ {s\theta_{3} } & 0 & {c\theta_{3} } & {l_{3} s\theta_{3} } \\ 0 & { - 1} & 0 & 0 \\ 0 & 0 & 0 & 1 \\ \end{array} } \right]; \, {}_{3}^{4} T{ = }\left[ {\begin{array}{*{20}c} {c\theta_{4} } & 0 & {s\theta_{4} } & {l_{4} c\theta_{4} } \\ {s\theta_{4} } & 0 & { - c\theta_{4} } & {l_{4} s\theta_{4} } \\ 0 & 1 & 0 & 0 \\ 0 & 0 & 0 & 1 \\ \end{array} } \right]; \hfill \\ {}_{4}^{5} T{ = }\left[ {\begin{array}{*{20}c} {c\theta_{5} } & 0 & { - s\theta_{5} } & {l_{5} c\theta_{5} } \\ {s\theta_{5} } & 0 & {c\theta_{5} } & {l_{5} s\theta_{5} } \\ 0 & { - 1} & 0 & 0 \\ 0 & 0 & 0 & 1 \\ \end{array} } \right]; \, {}_{5}^{6} T{ = }\left[ {\begin{array}{*{20}c} {c\theta_{6} } & { - s\theta_{6} } & 0 & {l_{6} c\theta_{6} } \\ {s\theta_{6} } & {c\theta_{6} } & 0 & {l_{6} s\theta_{6} } \\ 0 & 0 & 1 & 0 \\ 0 & 0 & 0 & 1 \\ \end{array} } \right]; \hfill \\ {}_{6}^{7} T{ = }\left[ {\begin{array}{*{20}c} {c\theta_{7} } & { - s\theta_{7} } & 0 & {l_{7} c\theta_{7} } \\ {s\theta_{7} } & {c\theta_{7} } & 0 & {l_{7} s\theta_{7} } \\ 0 & 0 & 1 & {d_{7} } \\ 0 & 0 & 0 & 1 \\ \end{array} } \right]. \hfill \\ \end{gathered} $$

The FK equation of the end-effector relative to the base is produced by multiplying all homogeneous transformation matrices, as shown in Eq. (24).

24 $$ T_{{\text{End - Effector}}} = {}_{0}^{7} T = {}_{0}^{1} T \cdot {}_{1}^{2} T \cdot {}_{2}^{3} T \cdot {}_{3}^{4} T \cdot {}_{4}^{5} T \cdot {}_{5}^{6} T \cdot {}_{6}^{7} T $$

where $T_{{\text{End - Effector}}}$ represents the end-effector's homogeneous transformation matrix with regard to the base coordinate system. When the value of a given joint variable is in Eq. (24), the alternative representation of $T_{{\text{End - Effector}}}$ can be written as Eq. (25).

25 $$ T_{{\text{End - Effector}}} = \left[ {\begin{array}{*{20}l} {{\vec{\mathbf{n}}}} \hfill & {{\vec{\mathbf{s}}}} \hfill & {{\vec{\mathbf{a}}}} \hfill & {{\vec{\mathbf{p}}}} \hfill \\ 0 \hfill & 0 \hfill & 0 \hfill & 1 \hfill \\ \end{array} } \right] = \left[ {\begin{array}{*{20}c} {n_{x} } & {s_{x} } & {a_{x} } & {p_{x} } \\ {n_{y} } & {s_{y} } & {a_{y} } & {p_{y} } \\ {n_{z} } & {s_{z} } & {a_{z} } & {p_{z} } \\ 0 & 0 & 0 & 1 \\ \end{array} } \right] $$

where $(p_{x} ,p_{y} ,p_{z} )^{{\text{T}}}$ represents the end-effector's position element in the base coordinate system, and $({\vec{\mathbf{n}}},{\vec{\mathbf{s}}},{\vec{\mathbf{a}}})$ represents the posture element, that is, the rotation element.

Although the rotation matrix $({\vec{\mathbf{n}}},{\vec{\mathbf{s}}},{\vec{\mathbf{a}}})$ has nine elements, it has only three degrees of freedom and is a unit orthogonal matrix with redundancy. Therefore, the Euler angle is used to describe the posture of the end-effector, and its calculation formula is shown in Eq. (26)^66^.

26 $$ \left( {\alpha ,\beta ,\gamma } \right) = \left( {\arctan \left( { - {{a_{y} } \mathord{\left/ {\vphantom {{a_{y} } {a_{z} }}} \right. \kern-\nulldelimiterspace} {a_{z} }}} \right),\arctan \left( {{{a_{x} } \mathord{\left/ {\vphantom {{a_{x} } {\sqrt {n_{x}^{2} + s_{x}^{2} } }}} \right. \kern-\nulldelimiterspace} {\sqrt {n_{x}^{2} + s_{x}^{2} } }}} \right),\arctan \left( { - {{s_{x} } \mathord{\left/ {\vphantom {{s_{x} } {n_{x} }}} \right. \kern-\nulldelimiterspace} {n_{x} }}} \right)} \right) $$

Thus, the position and posture can be expressed as $P = (p_{x} ,p_{y} ,p_{z} ,\alpha ,\beta ,\gamma )$, where $(p_{x} ,p_{y} ,p_{z} )$ is the position vector and $(\alpha ,\beta ,\gamma )$ is the posture vector expressed by Euler angle. The FK equation of the simplified 7-DOF robotic manipulator is shown in Eq. (27).

27 $$ \begin{gathered} p_{x} = d_{7} (s_{5} h - c_{5} b) + l_{7} s_{7} o + n(l_{6} + l_{7} c_{7} ) - l_{5} i - l_{4} h - l_{3} a + l_{2} c_{12} \hfill \\ p_{y} = - d_{7} (s_{5} j - c_{5} d) - l_{7} s_{7} (c_{6} k + s_{6} p) - (l_{6} + l_{7} c_{7} )(s_{6} k - c_{6} p) + l_{5} p + l_{4} j + l_{3} c + l_{2} c_{2} s_{1} \hfill \\ p_{z} = d_{7} l + l_{7} s_{7} (s_{6} m - c_{6} f) - (l_{6} + l_{7} c_{7} )(c_{6} m + s_{6} f) - l_{5} m - l_{4} e - l_{3} c_{3} s_{2} - l_{2} s_{2} + l_{1} \hfill \\ \alpha = {\text{atan}} ((s_{5} j - c_{5} d)/l) \hfill \\ \beta = {\text{atan}} ((s_{5} h - c_{5} b)/((c_{7} n + s_{7} o)^{2} + (c_{7} o - s_{7} n)^{2} )^{0.5} ) \hfill \\ \gamma = {\text{atan}} ((s_{7} n - c_{7} o)/(c_{7} n + s_{7} o)) \hfill \\ {\text{where }}a = s_{13} - c_{123} \, b = c_{3} s_{1} + c_{12} s_{3} , \, c = c_{1} s_{3} + c_{23} s_{1} , \, d = c_{13} - c_{2} s_{13} , \hfill \\ \, e = c_{2} s_{4} + c_{34} s_{2} , \, f = c_{24} - c_{3} s_{24} , \, g = s_{4} a - c_{14} s_{2} , \, h = c_{4} a + c_{1} s_{24} , \hfill \\ \, i = c_{5} h + s_{5} b, \, j = c_{4} c - s_{124} \, k = s_{4} c + c_{4} s_{12} , \, l = s_{5} e + c_{5} s_{23} , \hfill \\ \, m = c_{5} e - s_{235} , \, n = s_{6} g - c_{6} i, \, o = c_{6} g + s_{6} i, \, p = c_{5} k + s_{5} d. \hfill \\ \end{gathered} $$

where $s_{i}$ and $c_{i}$ stand in for $\sin (\theta_{i} )$ and $\cos (\theta_{i} )$, $s_{ij}$ and $c_{ij}$ stand in for $\sin (\theta_{i} ) \cdot \sin (\theta_{j} )$ and $\cos (\theta_{i} ) \cdot \cos (\theta_{j} )$, respectively.

As mentioned above, the FK equation of the 7-DOF robotic manipulator can be easily obtained by using DH coordinate method. Given the joint angle vector $\left( {\theta_{1} ,\theta_{2} ,\theta_{3} ,\theta_{4} ,\theta_{5} ,\theta_{6} ,\theta_{7} } \right)$, the position and posture $(p_{x} ,p_{y} ,p_{z} ,\alpha ,\beta ,\gamma )$ of the manipulator can be directly calculated by Eq. (27). However, given the position and posture $(p_{x} ,p_{y} ,p_{z} ,\alpha ,\beta ,\gamma )$ of the manipulator, the IK equation used to obtain the joint angle vector $\left( {\theta_{1} ,\theta_{2} ,\theta_{3} ,\theta_{4} ,\theta_{5} ,\theta_{6} ,\theta_{7} } \right)$ is highly nonlinear, which is considered to be a very challenging optimization problem^76^.

## EOSMA for inverse kinematics

The manipulator's IK problem is defined as determining the corresponding joint angle based on the position and posture of the end-effector. The IK problem of complex structure manipulator belongs to the NP problem group^83^. Due to the analytical method is extremely difficult to use, this research employs the developed EOSMA to address the IK problem. The relationship between the EOSMA algorithm and the IK problem is shown in Table 2.

**Table 2:** The correspondence between EOSMA and IK problem.

Biological principle	EOSMA	IK problem
Slime mould location	Search agent location *pBest*	Candidate joint angles of the manipulator
The venous form of slime mould	Global optimum location *gBest*	The best joint angle
Food odor concentration	Fitness value *S*	The error between the end-effector poses corresponding to the candidate joint angles and the desired pose
Positive and negative feedback	Search agent location weights *W*	Weight of candidate joint angles
Transition contraction mode	Adaptive parameter *z*	Update method of candidate joint angles
Close to or away from food sources	Adaptive parameter *vb*	Update direction of candidate joint angles

The purpose of this study is to optimize the joint angle vector $\overrightarrow {{\theta_{i} }} = (\theta_{1} ,\theta_{2} ,\theta_{3} ,\theta_{4} ,\theta_{5} ,\theta_{6} ,\theta_{7} )$ of the manipulator to eliminate position and posture errors. The FK formula is used to calculate the end-effector’s position and posture corresponding to the joint angle vector. For the desired pose $P_{0} = (p_{x0} ,p_{y0} ,p_{z0} ,\alpha_{0} ,\beta_{0} ,\gamma_{0} )$, the fitness of the candidate joint angle vector $\overrightarrow {{\theta_{i} }}$ is defined as Eq. (28).

28 $$ f_{error} (\vec{\theta }_{i} ) = w_{1} \cdot f_{1} (\vec{\theta }_{i} ) + w_{2} \cdot f_{2} (\vec{\theta }_{i} ) $$

29 $$ f_{1} (\vec{\theta }_{i} ) = [(p_{xi} - p_{x0} )^{2} + (p_{yi} - p_{y0} )^{2} + (p_{zi} - p_{z0} )^{2} ]^{{\tfrac{1}{2}}} $$

30 $$ f_{2} (\vec{\theta }_{i} ) = [(\alpha_{i} - \alpha_{0} )^{2} + (\beta_{i} - \beta_{0} )^{2} + (\gamma_{i} - \gamma_{0} )^{2} ]^{{\tfrac{1}{2}}} $$

where $w_{1} + w_{2} = 1$ represents the weight of position and posture error, and $P_{i} = (p_{xi} ,p_{yi} ,p_{zi} ,\alpha_{i} ,\beta_{i} ,\gamma_{i} )$ denotes the end-effector’s position and posture corresponding to the joint angle vector $\overrightarrow {{\theta_{i} }}$, which can be obtained from Eq. (27).

The fitness function defined by Eq. (28) consists of position and posture error. It should be noted that in previous studies, many researchers only considered position without considering posture, reducing the complexity of the IK problem. Although those algorithms have obtained high accuracy, they are inconsistent with many real-world applications. The end-effector’s position and posture are considered comprehensively in this study, and the complete pose of the manipulator is obtained. For EOSMA, the location of the search agents is the joint angle vector, i.e., $\overrightarrow {{pBest_{i} }} = \overrightarrow {{\theta_{i} }}$. The search range of joint angles is presented in Table 1.

**Figure 6:**
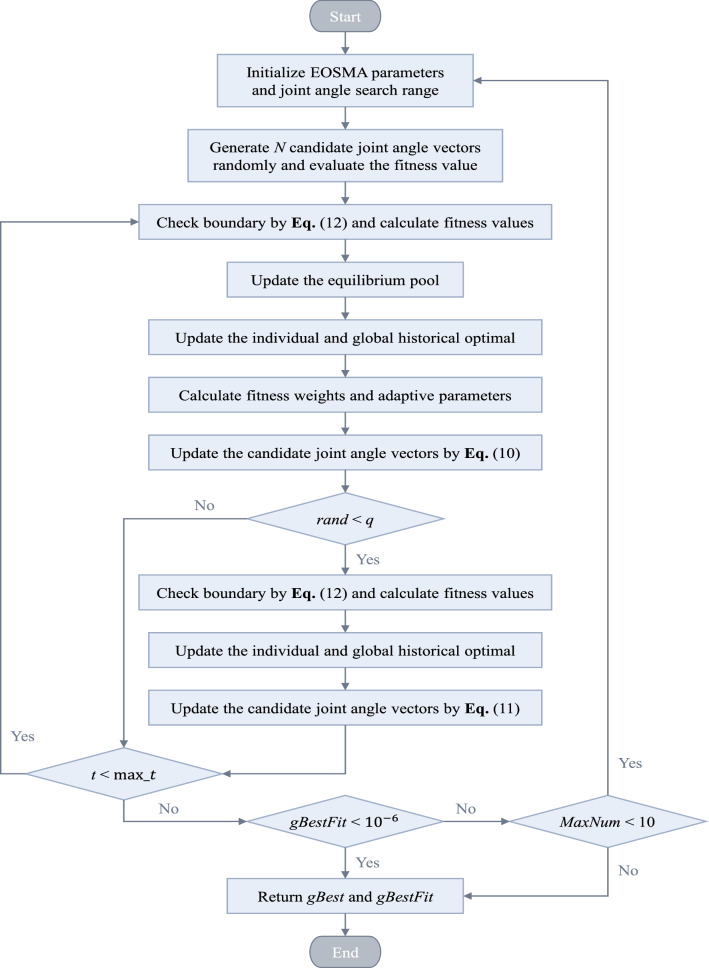
Flow chart of the EOSMA implementation for the IK problem.

Due to the randomness of the metaheuristic algorithm, poor outliers may appear in a single run, which will affect the average solution accuracy of the algorithm. In this study, the threshold for judging whether the algorithm has been solved successfully is set as 10e−6. If the solution result is less than 10e−6, the algorithm is considered to have been solved successfully, and the solution result of the algorithm is retained; Otherwise, the algorithm is employed to solve again until the algorithm's maximum number of failures is reached. The maximum number of failures of all comparison algorithms is set to 10. Figure 6 explains the flow chart of EOSMA for the IK problem.

## Experimental results and discussions

The effectiveness and efficiency of the EOSMA in handling the IK problem were validated in two scenarios in this section. Firstly, EOSMA was compared with 15 well-known algorithms without considering posture and then compared with the results of existing studies. Then, the proposed method was compared with 15 well-known single-objective algorithms and 9 multi-objective algorithms in the scenario of comprehensively considering position and posture. Finally, according to the calculated joint angle vector and the current angles of the manipulator, the joint change of the manipulator was simulated, and the motion trajectory of the end-effector was drawn. All algorithm codes were run in MATLAB R2020b, and the hardware details were Intel(R) Core (TM) i7-9700 CPU (3.00 GHz) and 16 GB RAM. In the experiment, the pose error and calculation time are given priority, and the best, worst, mean, and standard deviation are employed as the algorithm's performance metrics.

### Parameter settings

To fully demonstrate the effectiveness and efficiency of EOSMA in solving the IK problem, it is compared with 15 single-objective algorithms and 9 multi-objective algorithms. These algorithms include SMA^15^, EO^39^, DE^44^, TLBO^46^, FPA^43^, MRFO^40^, MPA^42^, PFA^42^, GBO^45^, HHO^47^, IGWO^48^, PSOGSA^49^, CODE^50^, MTDE^51^, SASS^52^, MOSMA^35^, MOPSO^53^, MOMPA^54^, MOALO^55^, MODA^56^, MOGWO^57^, MOMVO^58^, MSSA^59^, MOEA/D^60^. All algorithms use the same common parameters for a fair comparison, and other parameters are taken from the values suggested in the original paper, as shown in Tables 3 and 4. In scenario 1, the mutation probability $q$ of EOSMA is set as 0, and the exploration factor $a_{1}$ is set as 1. In scenario 2, the mutation probability $q$ is set as 1, and the exploration factor $a_{1}$ is set as 2.

**Table 3:** Parameter settings of the single-objective algorithms. For scenario 1, *N* = 50, *Max_t* = 500; For scenario 2, *N* = 100, *Max_t* = 1000.

Algorithms	Parameters	Values	Algorithms	Parameters	Values
EOSMA	Hybrid parameter *z*	0.5	IGWO	Convergence factor *a*	[2, 0]
	Mutation probability *q*	0 and 1	PSOGSA	Inertia weight *w*	[1, 0]
	Control volume *V*	1		Personal cognition coefficient *c*_1_	0.5
	Generation probability *GP*	0.5		Social cognition coefficient *c*_2_	1.5
	Exploration factor *a*_1_	1 and 2		Gravitational constant *G*_0_	1
	Exploitation factor *a*_2_	2		Constant *α*	23
SMA	Constant *z*	0.03	CODE	Scale factor *F*	0.5
EO	Control volume *V*	1		Crossover rate *Cr*	0.9
	Generation probability *GP*	0.5		Generation jumping rate *Jr*	0.3
	Exploration factor *a*_1_	2	MTDE	Constant *WinIter*	20
	Exploitation factor *a*_2_	1		Constant *H*	5
MRFO	Somersault factor *S*	2		Constant *initial*	0.001
MPA	Constant *p*	0.5		Constant *final*	2
	Constant *FADs*	0.2		Parameter *Mu*	log(*Dim*)
FPA	Scale factor *a*	2		Constant *μf*	0.5
	Constant *b*	0.5		Constant *σ*	0.2
	Proximity probability *p*	0.2	SASS	Constant *pr*	0.11
DE	Scale factor *F*	0.5		Population size *N*	[18*Dim, 4]
	Crossover rate *Cr*	0.9		Rank of diagonal matrix *rd*	0.5
GBO	Constant *pr*	0.5		Scale factor *c*	0.7
TLBO	Teaching factor *TF*	{1, 2}		Archiving size *Ar*	1.4
HHO	Constant *β*	1.5		Memory size *Ms*	100

**Table 4:** Parameter settings of the multi-objective algorithms. For all algorithms, archive size was set to 100, *N* = 100, *Max_t* = 1000.

Algorithms	Parameters	Values	Algorithms	Parameters	Values
MOEOSMA	Hybrid parameter *z*	0.5	MOPSO	Inertia weight *w*	0.5
	Mutation probability *q*	1		Damping rate	0.99
	Control volume *V*	1		Personal cognition coefficient *c*_1_	1
	Generation probability *GP*	0.5		Social cognition coefficient *c*_2_	2
	Exploration factor *a*_1_	2		Number of grids	7
	Exploitation factor *a*_2_	2		Grid inflation rate *α*	0.1
MOSMA	Constant *z*	0.03		Leader selection pressure *β*	2
MOMPA	Constant *p*	0.5		Deletion selection pressure *γ*	2
	Constant *FADs*	0.2		Mutation rate *μ*	0.1
MOGWO	Grid inflation rate *α*	0.1	MODA	Inertia weight *w*	0.9–0.7
	Number of grids *n*	10	MOMVO	Minimum probability *WEP*_min_	0.2
	Leader selection pressure *β*	4		Maximum probability *WEP*_max_	1
	Deletion selection pressure *γ*	2	MOEA/D	Crossover parameter *γ*	0.5
MOALO	Parameter less	NA	MSSA	Parameter less	NA

### Result obtained for scenario 1

#### Comparison of EOSMA with other SI algorithms

**Figure 7:**
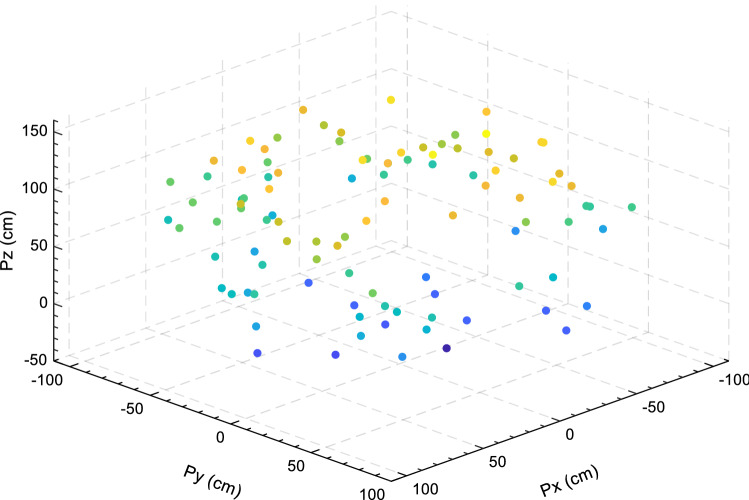
Randomly selected position points in the workspace of the manipulator.

In this part, EOSMA is compared against 15 well-known algorithms for the IK problem that do not take posture into account. Due to the metaheuristic algorithms run at random, each run will have a higher or lower value than the preceding one. To avoid the influence of randomness in the selection of position points, 100 different position points were generated at random in the workspace of the manipulator, as shown in Fig. 7, where the color represent the position's height.

The results obtained by the comparison algorithm are shown in Table 5. It can be seen that EOSMA, EO, MRFO, PFA, and GBO can all obtain theoretical optimal solutions with zero error without considering the posture, but EOSMA has the best robustness and the shortest solution time. The average convergence accuracy of EOSMA is 9 orders of magnitude higher than EO and 13 orders of magnitude higher than SMA, which verifies the effectiveness and efficiency of EOSMA in the IK problem.

**Table 5:** Comparative results of inverse kinematics problem.

Algorithm	EOSMA	SMA	EO	MRFO	MPA	PFA	FPA	DE
Worst	**7.85E−16**	3.09E−02	7.99E−07	9.92E−07	4.28E−07	2.66E−07	5.99E−02	9.93E−07
Mean	**2.64E−17**	9.57E−04	2.45E−08	1.37E−07	7.75E−08	5.73E−09	6.57E−03	2.97E−07
Best	**0.00E+00**	4.17E−05	**0.00E+00**	**0.00E+00**	1.43E−08	**0.00E+00**	3.26E−12	5.00E−14
Std	**9.93E−17**	3.27E−03	1.06E−07	2.27E−07	6.73E−08	3.43E−08	9.19E−03	2.80E−07
Time(s)	**0.0567**	0.5382	0.0649	0.1302	0.1284	0.0812	1.2808	0.2809

Algorithm	GBO	TLBO	HHO	IGWO	PSOGSA	CODE	MTDE	SASS
Worst	1.10E−11	2.42E−03	3.99E−02	1.35E−02	3.13E−13	2.76E−01	6.15E−03	1.01E−03
Mean	2.54E−13	2.23E−04	1.64E−03	2.84E−03	1.57E−13	5.79E−02	1.07E−03	1.99E−04
Best	**0.00E+00**	9.12E−09	9.03E−12	3.86E−05	4.37E−14	3.11E−03	1.27E−05	7.04E−11
Std	1.48E−12	4.56E−04	4.54E−03	3.26E−03	6.78E−14	5.71E−02	1.16E−03	2.28E−04
Time(s)	0.3058	1.6861	1.7444	5.5963	0.2084	0.4410	2.1214	1.2234

The optimal values are shown in bold.

**Figure 8:**
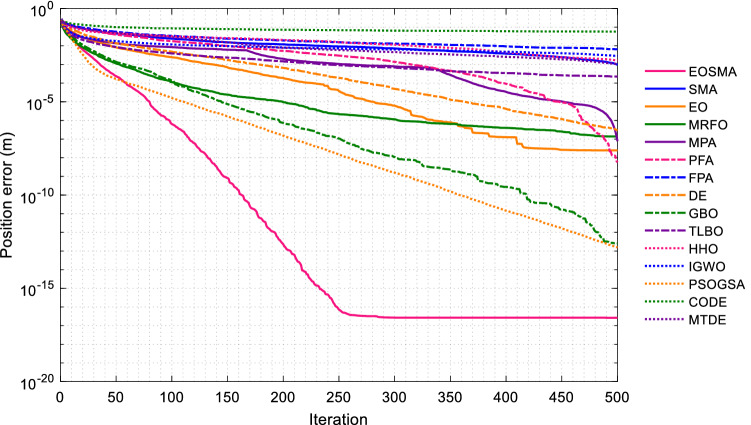
Average convergence curve of randomly selected position points.

Convergence curves of EOSMA and 14 comparison algorithms are shown in Fig. 8. Since the population size of SASS decreases linearly with the number of iterations, its convergence curves are not comparable. The results show that EOSMA can quickly obtain high-precision solutions, far superior to other comparison algorithms, followed by GBO and PSOGSA, indicating that EOSMA is suitable for solving IK problem without considering posture.

**Figure 9:**
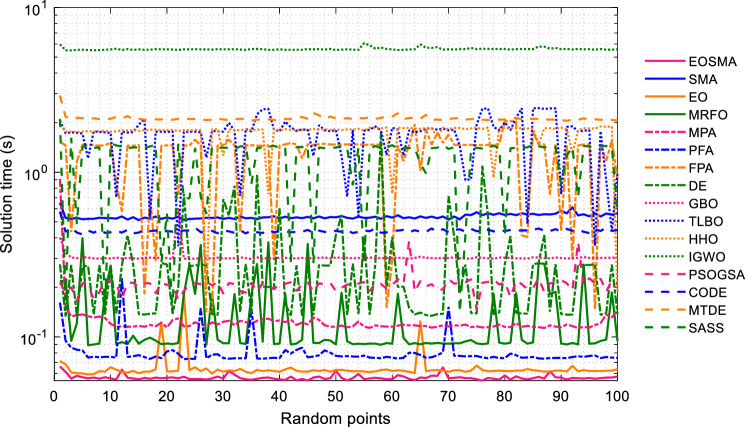
Solution time of comparison algorithms at randomly selected position points.

The solution time of EOSMA and 15 comparison algorithms at 100 randomly selected positions is shown in Fig. 9. It can be seen that EOSMA takes the least amount of time, followed by EO and PFA, and IGWO takes the most time. Since the manipulator is a real-time control system, the algorithm with a short solution time is preferred when the solution accuracy is satisfied. Therefore, although PSOGSA and GBO have high convergence accuracy, they are not suitable for solving the IK of the manipulator. EOSMA, EO, and PFA are highly competitive in the IK problem.

**Figure 10:**
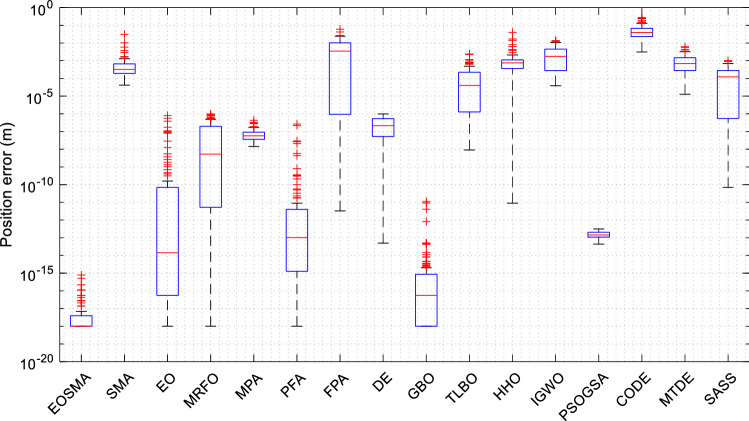
Box plot of optimization results of randomly selected position points.

Figure 10 shows the distribution of the solution results of the algorithm in the form of the box plot. For the convenience of observation, set results less than 10e−18 to 10e−18. It is clear that EOSMA has a lower median and a narrower box plot with fewer outliers than most algorithms. EOSMA is superior to SMA in convergence accuracy and EO in robustness.

**Figure 11:**
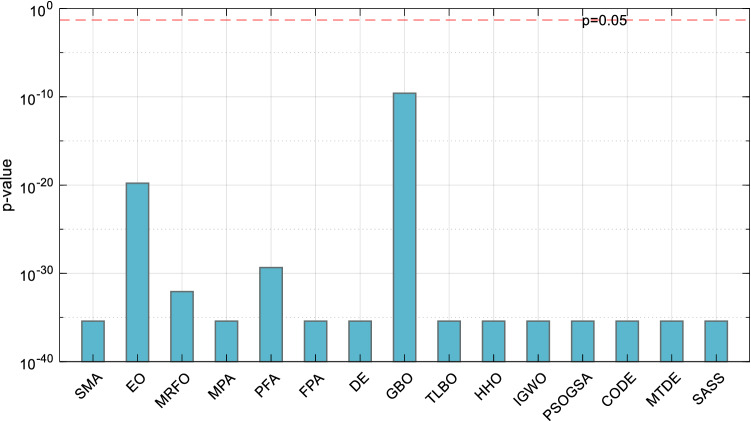
Wilcoxon rank-sum test results of randomly selected position points.

To verify whether there is a significant difference between the solution results of EOSMA and each comparison algorithm, the Wilcoxon rank-sum test of two paired samples was utilized^84^. Figure 11 illustrates the *p*-value of the Wilcoxon rank-sum test as a bar graph. If *p* < 0.05, it is believed that there is a substantial difference between the two algorithms. As can be seen, EOSMA differs greatly from all comparison algorithms, particularly SMA, indicating that the improvement is effective.

#### Comparison of EOSMA with the existing studies

Many metaheuristic algorithms, such as quantum particle swarm optimization (QPSO)^9^, GWO^74^, and WOA^75^, have been effectively applied to the IK of 7-DOF robotic manipulators. Table 6 shows the results of EOSMA, SMA, and EO in the IK of 7-DOF manipulator with other comparable metaheuristic algorithms used in existing studies. It is clear from the results that the solution accuracy of EOSMA is 4 orders of magnitude higher than that of QPSO.

**Table 6:** Comparative results of inverse kinematics problem.

Algorithm	Swarm size	Position error (MSE)	Algorithms	Swarm size	Position error (MSE)
PSO^9^	300	2.1162E−04	WOA^75^	50	9.5460E−04
ABC^9^	100	1.1105E−06	SMA	50	9.5688E−04
FA^9^	50	1.4547E−05	EO	50	2.4514E−08
QPSO^9^	150	6.9347E−13	EOSMA	50	2.6428E−17
GWO^74^	50	9.4745E−08			

### Result obtained for scenario 2

#### Single objective optimization

**Figure 12:**
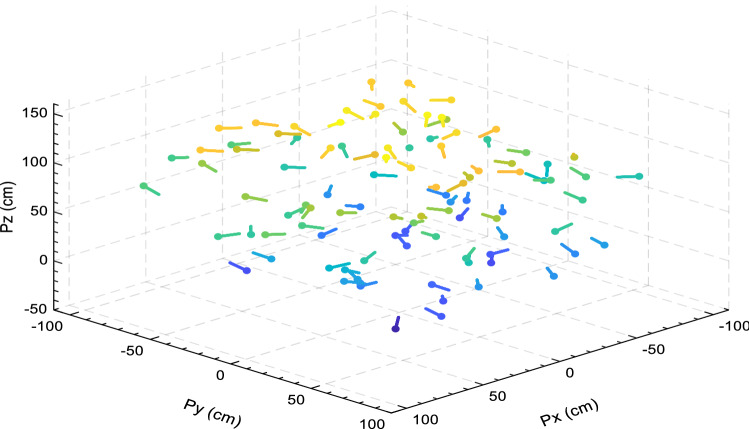
Randomly generated pose points in the workspace of the manipulator.

The IK of redundant manipulators is considered a challenging optimization problem^83^. Many previous studies did not consider the end-effector’s posture, which simplifies the problem but is inconsistent with most practical applications. Considering that the posture makes the IK problem more complex, so it is necessary to verify EOSMA's optimization performance further. The linear weighting method is utilized in this section to handle the IK problem while keeping posture in consideration. The fitness value of the candidate joint angle vector $\overrightarrow {{\theta_{i} }}$ is calculated by Eq. (28), where $w_{1}$ and $w_{2}$ are set to 0.5, indicating that position and posture are equally important. A total of 100 different pose points were randomly generated in the workspace of the 7-DOF manipulator, as shown in Fig. 12. In the figure, solid dots represent the position of the end-effector, and straight lines represent the posture.

Table 7 presents the results of EOSMA and 15 comparison algorithms. When considering the end-effector’s position and posture, it can be seen that only EOSMA, MPA, DE, and SASS can effectively solve the IK problem. EOSMA and SASS produced acceptable results, with an average solution accuracy of 10e-18. Although EOSMA's solution accuracy is not as good as SASS's, its solution time is shorter, making it more suitable for manipulator real-time control. As a result, EOSMA is a viable alternative method for solving the IK problem of complicated manipulators.

**Table 7:** Optimization results take into account the posture of the end-effector.

Algorithm	EOSMA	SMA	EO	MRFO	MPA	PFA	FPA	DE
Worst	**5.55E−17**	0.242342	0.289362	0.26697	9.71E−07	0.23774	0.230746	1.28E−12
Mean	7.79E−18	0.043938	0.02786	0.009398	4.55E−07	0.020017	0.037691	1.04E−13
Best	**0.00E+00**	0.001052	5.82E−11	1.99E−16	4.01E−08	2.95E−13	6.58E−05	5.26E−16
Std	1.21E−17	0.050096	0.047801	0.035825	2.54E−07	0.044404	0.045514	1.95E−13
Time (s)	**0.360804**	1.680179	2.207461	1.395682	0.424897	2.057285	9.616893	0.896265

Algorithm	GBO	TLBO	HHO	IGWO	PSOGSA	CODE	MTDE	SASS
Worst	0.229208	0.053045	0.473011	0.101893	0.410281	0.117581	0.016585	8.09E−17
Mean	0.007624	0.004314	0.147295	0.0027	0.07595	0.027037	0.000283	**5.35E−18**
Best	**0.00E+00**	8.53E−11	0.005506	0.000123	1.76E−13	0.005138	1.85E−09	**0.00E+00**
Std	0.035906	0.009956	0.10784	0.010879	0.091469	0.0198	0.002008	**1.06E−17**
Time (s)	4.995488	5.640442	8.849817	22.94664	12.72971	7.604563	2.485424	0.61688

The optimal values are shown in bold.

**Figure 13:**
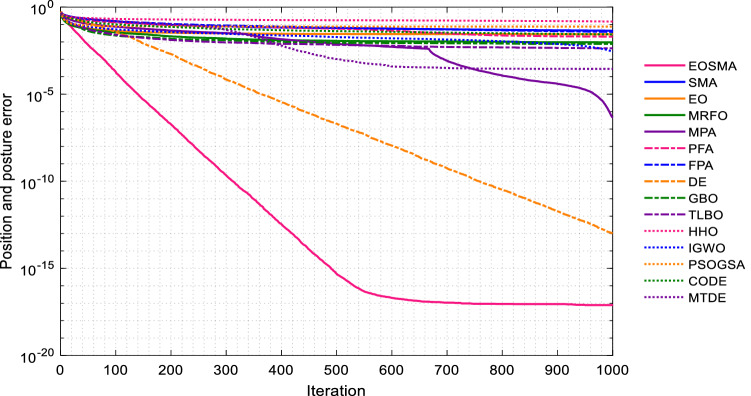
Average convergence curve of randomly generated pose points.

Figure 13 presents the convergence curves of EOSMA and various comparison algorithms. As can be seen, EOSMA has the fastest convergence speed and the highest convergence accuracy, considerably outperforming EO and SMA. Furthermore, the convergence curve of EOSMA is remarkably smooth, indicating that the algorithm has achieved a reasonable balance between exploration and exploitation. The random difference mutation operator is used in EOSMA to expand the search space of search agents during the iterative process, avoid overcrowding of search agents, and increase the probability of finding the optimal solution. As shown in Fig. 13, the average solution accuracy of most algorithms is less than 10e-7, indicating that the proposed EOSMA improves the average solution accuracy by 10 orders of magnitude.

**Figure 14:**
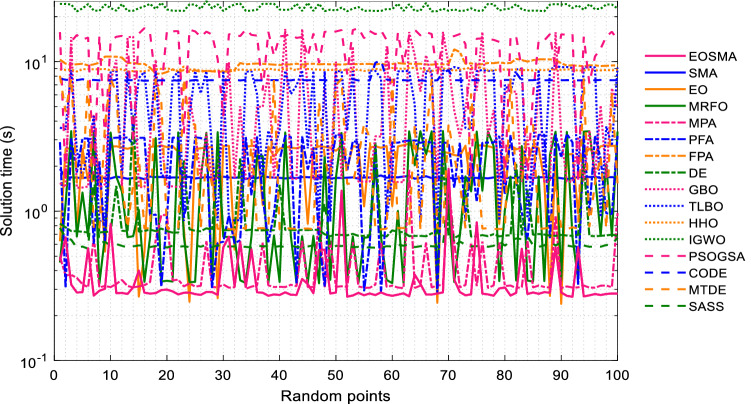
Solution time of comparison algorithms at randomly generated pose points.

The solution time of each algorithm at 100 pose points is shown in Fig. 14. It can be seen that the solution time of EOSMA fluctuates little when solving different pose points. The average solution time of EOSMA is the shortest, about 0.36 s, followed by MPA, about 0.42 s. This may not be an entirely satisfactory result, but it shows that EOSMA can still be used for some robotic manipulators with low real-time performance, such as in the service industry and offline computing online operations^66^.

**Figure 15:**
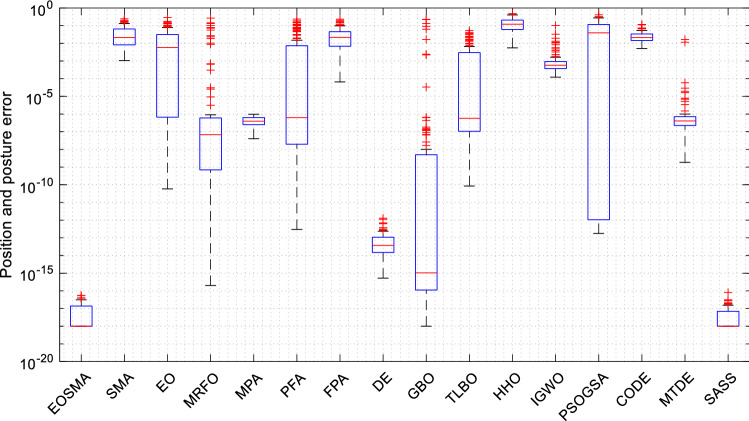
Box plot of optimization results of randomly generated pose points.

The box plot in Fig. 15 displays the solution outcomes of EOSMA and other comparison algorithms at 100 pose points. EOSMA and SASS have the lowest median and few outliers, making them considerably superior to other comparison algorithms. Overall, EOSMA and SASS performed well on the IK problem, with little difference in performance between the two. However, EOSMA has a lower time complexity than SASS.

**Figure 16:**
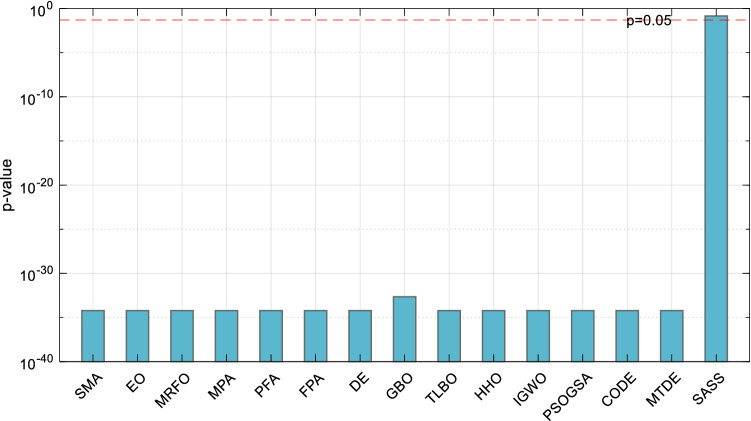
Wilcoxon rank-sum test results of randomly generated pose points.

Figure 16 shows the Wilcoxon *p*-value test results of EOSMA and each comparison algorithm. It can be seen that, except for SASS, there are significant differences between the optimization results of EOSMA and comparison algorithms at the confidence level of 0.05. It shows that the search principle of EOSMA is different from other algorithms and can solve the IK more effectively.

#### Multi objective optimization

**Figure 17:**
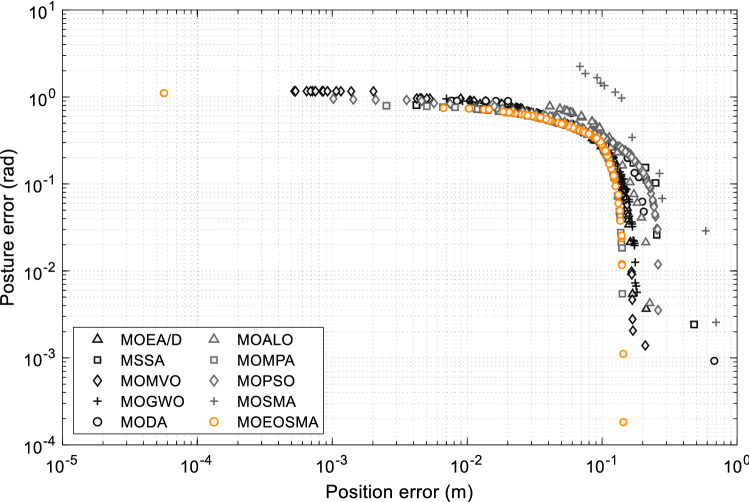
The PF obtained by multi-objective algorithms at desired pose *P*_1_.

**Figure 18:**
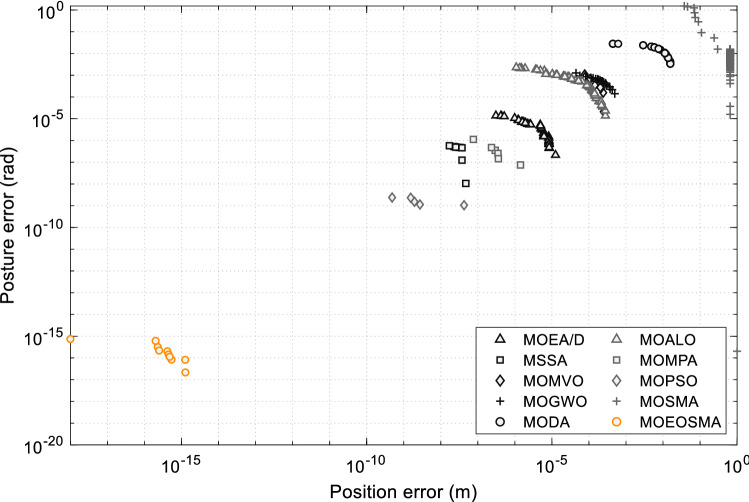
The PF obtained by multi-objective algorithms at desired pose *P*_2_.

If the desired position and posture of the end-effector are considered comprehensively, there may be no inverse kinematics solution due to the structural restrictions of the manipulator, that is, the position and posture errors cannot be optimized simultaneously. As a result, the manipulator's IK can be regarded as a multi-objective optimization problem. Obviously, the closer to the workspace boundary, the less selectable posture of the end-effector. In this case, it is difficult to obtain a satisfactory solution using the single-objective algorithm. In this study, MOEOSMA was proposed to solve IK problems. The desired pose $P_{1} = ( - 25,100,50,0,0,0)$ and $P_{2} = (50, - 25,75,0,0,0)$ were selected as test cases. It was verified that $P_{1}$ did not have inverse kinematic solutions while $P_{2}$ had inverse kinematic solutions through the Robotics Toolbox for MATLAB. Due to the IK problem of the 7-DOF manipulator has not been studied using the multi-objective method in the previous literature, MOEOSMA is compared with MOSMA^35^, MOPSO^53^, MOMPA^54^, MOALO^55^, MODA^56^, MOGWO^57^, MOMVO^58^, MSSA^59^, and MOEA/D^60^. For a fair comparison, the population size of all algorithms was set to 100, the maximum number of iterations was set to 1000, the archive size was set to 100, and each example was independently run 20 times. Since the true PF is unknown, the hypervolume (HV) metric^85,86^ was used to evaluate the performance difference of the algorithms. The HV metric can evaluate both the advancement and distribution of the obtained PF simultaneously^87^. The larger HV value, the better convergence and distribution of the algorithm. The reference points for the test cases used in this study were 1.1 times the maximum objective function value found in all algorithms and all optimization runs. The reference points for calculating the HV values of the desired poses *P*_1_ and *P*_2_ are (2.088567, 2.466816) and (1.695669, 2.524178), respectively. Table 8 provides the statistical data of the HV results obtained by each algorithm. The PF obtained by the algorithms under the two desired poses is shown in Figs. 17 and 18, respectively.

**Table 8:** HV results of multi-objective algorithms on two desired poses.

Pose	Index	MOEOSMA	MOSMA	MOPSO	MOMPA	MOALO	MODA	MOGWO	MOMVO	MSSA	MOEA/D
*P*_1_	Mean	**5.07631**	4.65143	5.00599	5.04500	4.86302	4.81951	4.94830	5.02802	5.00469	4.88152
	Std	**0.02007**	0.06950	0.04402	0.02733	0.09082	0.22506	0.08276	0.04602	0.05326	0.26105
	FR (Rank)	**1.40 (1)**	9.80 (10)	4.60 (4)	2.95 (2)	7.95 (9)	7.90 (8)	5.95 (6)	3.50 (3)	4.80 (5)	6.15 (7)
	Time (s)	5.87559	41.00294	28.82177	3.98753	25.23976	119.88871	138.56085	**3.60248**	5.12301	249.77852
*P*_2_	Mean	**4.28017**	4.00487	4.22408	4.27983	4.19377	4.16979	4.24412	4.25582	4.26781	4.22517
	Std	**3.17E-13**	0.05168	0.07739	0.00148	0.08250	0.09992	0.03762	0.02288	0.02584	0.08037
	FR (Rank)	**1.00 (1)**	9.70 (10)	5.75 (6)	2.80 (2)	7.10 (8)	8.10 (9)	5.45 (5)	5.30 (4)	3.60 (3)	6.20 (7)
	Time (s)	**0.81779**	38.63682	25.27879	1.41943	25.87101	152.13011	67.21598	2.35827	2.65555	229.49681

The optimal values are shown in bold, and FR stands for Friedman's Rank.

The data provided in Table 8 show that MOEOSMA obtains the best mean and standard deviation in the two scenarios, while MOMPA and MOMVO also show strong competition. For the pose without inverse kinematic solution, MOEOSMA has a longer solution time than MOMPA, MOMVO and MSSA, but the quality of PF obtained is better. For the pose with inverse kinematic solution, MOEOSMA is much better than the other comparison algorithms in both accuracy and speed. As can be seen from Fig. 17, the PF obtained by MOEOSMA is closer to the true PF, and extreme Pareto solutions are more widely distributed. As can be seen from Fig. 18, the PF of MOEOSMA is convex, and the rest is concave, indicating that the proposed algorithm can minimize both position and posture errors, while the other algorithms tend to optimize one of the objectives. This fully reveals that MOEOSMA is a good optimization tool for solving the IK problem of redundant manipulators.

### Simulation and test

The motion state of the 7-DOF robotic manipulator was simulated in this section by using the Robotics Toolbox for MATLAB. Assume the beginning joint angle vector of the manipulator is $\overrightarrow {{\theta_{1} }} = (45^\circ ,0^\circ ,45^\circ ,0^\circ ,45^\circ ,0^\circ ,0^\circ )$, the position and posture of the end-effector corresponding to the joint angle vector $\overrightarrow {{\theta_{1} }}$ is $P_{1} = ( - 24.748737,100.961941,50.000000,90, - 45,0)$, and the desired end-effector pose is $P_{2} = (50, - 75,75,0,0,0)$. According to the desired pose, many joint angle vectors can be obtained through EOSMA. For the current state of the manipulator, the cost of changing to those joint angles is different. In this study, the joint angle vector with the slightest overall angle change is the best candidate joint angle vector, which can minimize the movement time of the manipulator. The penalty function of joint angles difference was added into the fitness function to evaluate the pose error, as shown in Eq. (31).

31 $$ O(\vec{\theta }_{i} ) = f_{error} (\vec{\theta }_{i} ) + w \cdot \left\| {\vec{\theta }_{i} - \vec{\theta }_{1} } \right\| $$

where $O(\vec{\theta }_{i} )$ represents the objective function, $\vec{\theta }_{i}$ represents the *i*-th candidate joint angle vector, $\vec{\theta }_{1}$ represents the joint angle vector in the starting state, $w$ is the penalty coefficient, the value in this paper is 10e−15, and $\left\| \cdot \right\|$ represents the calculated Euclidean distance.

The joint angles of the manipulator obtained by EOSMA, SMA, and EO are shown in Table 9. The manipulator can read the starting and the ending joint angles from Table 9 to control the rotation of each joint angle and move the end-effector to the desired position and posture.

**Table 9:** Inverse solution results of the 7-DOF robotic manipulator.

Posture	Algorithms	*θ*_1_(°)	*θ*_2_(°)	*θ*_3_(°)	*θ*_4_(°)	*θ*_5_(°)	*θ*_6_(°)	*θ*_7_(°)	Distance
*P*_1_	–	45	0	45	0	45	0	0	–
*P*_2_	EOSMA	43.506898	− 90.000000	− 90.000000	− 38.841935	− 2.78E− 14	64.745704	20.589333	185.6794
	SMA	− 0.014719	− 90.000000	− 90.000000	24.224333	2.93E− 12	53.065842	12.725225	184.2310
	EO	− 174.758930	− 86.555831	79.782405	− 18.741263	10.776619	− 51.677450	− 14.648226	247.7969

**Figure 19:**
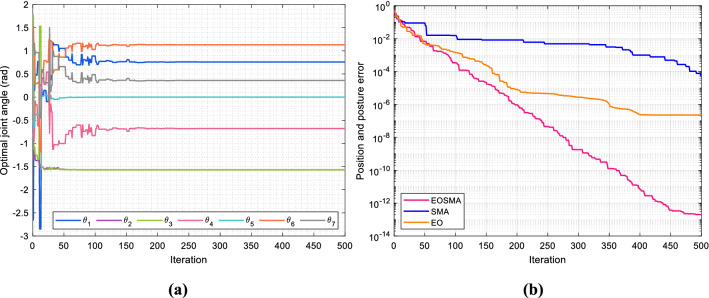
Optimization process of the algorithm. (**a**) Optimal candidate joint angle of EOSMA varies with the number of iterations. (**b**) Convergence curve of the algorithms.

**Figure 20:**
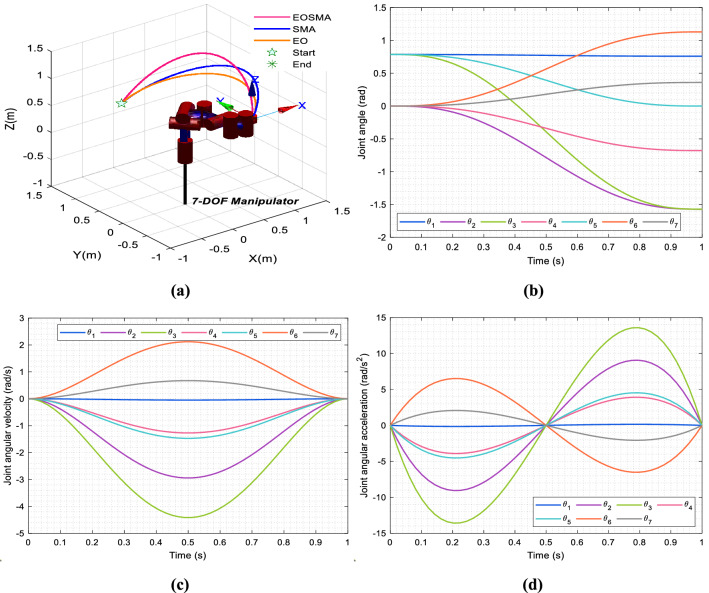
Simulation test results. (**a**) The trajectory of end-effector of the 7-DOF manipulator. (**b**) Curves of joint angle with time. (**c**) Curves of joint angular velocity with time. (**d**) Curves of joint angular acceleration with time.

Figure 19 shows the optimization process of EOSMA, Fig. 20 shows the trajectory of the end-effector and the curve of the joint angle change with time. The simulation results show that the three algorithms can reach the desired position and posture, and in which the angle change of SMA is the least, but the solution accuracy is the lowest. The angle change of EOSMA is very close to that of SMA, but the pose error is reduced by 8 orders of magnitude, as shown in Table 9 and Fig. 19b. EO has the largest angle variation, and its accuracy is between SMA and EOSMA. As shown in Fig. 19b, the optimal candidate joint angle does not exceed the search range of each joint angle during iteration. At the beginning of the iteration, the angle of each joint changed obviously, indicating that EOSMA has a strong exploration ability. After 200 generations, the optimal candidate joint angle did not change significantly. EOSMA used the SMA search operator to fine-adjust the optimal candidate joint angle found so far, achieving high convergence accuracy. As can be seen from Fig. 20a, all three algorithms obtain a very smooth trajectory, but EOSMA has the highest accuracy in reaching the desired pose. It can be seen from Fig. 20b–d that the angle, velocity, and acceleration curves of each joint are continuous and smooth, and the angle change of each joint is evenly distributed. It indicates that there is no obvious jitter during the movement of the manipulator, and the overall change range of the manipulator is small.

### Results discussion

The EOSMA proposed in this study enhances the search ability of EO and SMA, increases population variety, and reduces the probability of falling into the local optimum. In fact, the most important obstacle in many metaheuristic algorithms is frequently falling into local optimum, which dramatically limits the optimization performance. When evaluated from this perspective, EOSMA is ahead of many heuristic algorithms (Figs. 8 and 13). By comparing the convergence curves of the two scenarios, it can be found that many algorithms can obtain high precision solutions in the scenario without considering the posture. Only EOSMA, DE, and MPA can effectively solve the scenarios comprehensively considering the position and posture. It shows that it is difficult to solve the scenario considering posture, and many algorithms will fall into local optimum. On the contrary, DE obtains higher convergence accuracy in the scenario considering posture, which indicates that the IK problem considering posture requires the algorithm to have strong exploration ability. In contrast, the IK problem not considering posture requires the algorithm to have strong exploitation ability. Therefore, in scenario 1, the parameters of EOSMA were set as follows: exploration coefficient $a_{1} = 1$, exploitation coefficient $a_{2} = 2$, and mutation probability $q = 0$; In scenario 2, the parameters of EOSMA were set as: exploration coefficient $a_{1} = 2$, exploitation coefficient $a_{2} = 2$, and mutation probability $q = 1$.

Due to the performance of many algorithms in these two scenarios differing greatly, previous studies did not compare the two scenarios. EOSMA can be well adapted to the IK problem in different scenarios by simply adjusting the parameters that control exploration and exploitation abilities, demonstrating the hybrid EOSMA has a strong generalization ability. The excellent performance of EOSMA can be summed up as follows.

1. SMA has strong exploitation ability and EO exploration ability. The concentration update operator of EO was used to guide the global search of SMA to keep the balance between exploration and exploitation, increase population diversity and enhance the robustness and generalization ability.
2. The update operator of SMA in the exploitation stage is defective, and it is easy to guide the search agents to converge to the origin in the late iteration, resulting in an invalid search. The structure of SMA was simplified, the parameters and calculation time were reduced.
3. The greedy selection strategy was used to retain the individual historical optimal and global historical optimal locations and then update them based on the individual historical optimal and global historical optimal, which improves the search efficiency.
4. The random difference mutation strategy was included after upgrading the location of EOSMA to widen the search range of the search agents, enhance the possibility of search agents escaping from the local optimum, and avoid premature convergence.

Although the results of this study show that EOSMA and its multi-objective version outperform majority of comparison algorithms, it still has some limitations. There are many adjustable parameters in EOSMA. It may be difficult to set the parameters for different applications. It is necessary not only to know the influence of different parameters on the algorithm's exploration and exploitation but also some properties of the problem. In addition, the EOSMA proposed in this paper is designed for the IK problem and its effectiveness in other real-world problems needs to be further tested.

## Conclusions and future directions

In this paper, an EO-guided SMA was developed to improve search efficiency by widening the search range of slime mould in order to tackle the IK problem of redundant manipulators efficiently. The performance of EOSMA for the IK problem is verified by comparison with 15 single-objective and 9 multi-objective algorithms, and comparable algorithms used in previous studies. Without considering the posture, EOSMA is superior to 15 comparison algorithms in terms of best, worst, mean, standard deviation, and average solution time. EOSMA can converge to the global optimal with an average convergence accuracy of 10e−17 m, which is 4 orders of magnitude higher than the best comparison algorithm PSOGSA. The average solution time is about 0.05 s, and the robustness is the best. When considering position and posture, the performance of EOSMA is similar to SASS, but EOSMA has a shorter solution time. The average solution accuracy of EOSMA can reach 10e-18, and the average solution time is about 0.36 s. Compared with the 9 multi-objective optimization algorithms, EOSMA's multi-objective version obtains higher accuracy, more comprehensive coverage, and more uniform distribution of PF. Simulation results show that the overall change of joint angle obtained by EOSMA is small, and the motion trajectory is smooth without obvious jitter. Statistical results show that EOSMA has better performance for the IK problem, which is significantly different from other algorithms. For some desired poses, the position and posture errors cannot be eliminated synchronously and must be compromised between the two. MOEOSMA can provide users with a well-distributed PF to pick from, and it is an efficient alternative method for solving the IK problem of complicated manipulators. Although promising results have been achieved, there are still some problems that need to be studied in the future. EOSMA has many parameters and depending on the problem to be solved, parameter adaptive selection methods can be considered for the algorithm, such as parameter adaptive mechanism based on success history^88^ or parameter adaptive mechanism based on reinforcement learning^89^. In addition, EOSMA can be applied to other fields, such as photovoltaic parameter extraction and satellite posture adjustment.

## Data Availability

All data, models, and code generated or used during the study appear in the submitted article.
